# L-DOPA administration shifts the stability-flexibility balance towards attentional capture by distractors during a visual search task

**DOI:** 10.1007/s00213-022-06077-w

**Published:** 2022-02-11

**Authors:** P. Riedel, I. M. Domachowska, Y. Lee, P. T. Neukam, L. Tönges, S. C. Li, T. Goschke, M. N. Smolka

**Affiliations:** 1 Department of Psychiatry and Psychotherapy, Technische Universität Dresden, Fetscherstraße 74, 01307 Dresden, Germany; 2 Department of Psychology, Technische Universität Dresden, Zellescher Weg 17, 01069 Dresden, Germany; 3 Department of Neurology, Ruhr University Bochum, St. Josef-Hospital, Gudrunstraße 56, 44791 Bochum, Germany; 4 Centre for Tactile Internet With Human-in-the-Loop, Technische Universität Dresden, Georg-Schumman-Str. 9, 01187 Dresden, Germany

**Keywords:** Cognitive control, Stability-flexibility balance, Attentional capture, Attentional breadth, Dopamine

## Abstract

**Rationale:**

The cognitive control dilemma describes the necessity to balance two antagonistic modes of attention: stability and flexibility. Stability refers to goal-directed thought, feeling, or action and flexibility refers to the complementary ability to adapt to an ever-changing environment. Their balance is thought to be maintained by neurotransmitters such as dopamine, most likely in a U-shaped rather than linear manner. However, in humans, studies on the stability-flexibility balance using a dopaminergic agent and/or measurement of brain dopamine are scarce.

**Objective:**

The study aimed to investigate the causal involvement of dopamine in the stability-flexibility balance and the nature of this relationship in humans.

**Methods:**

Distractibility was assessed as the difference in reaction time (RT) between distractor and non-distractor trials in a visual search task. In a randomized, placebo-controlled, double-blind, crossover study, 65 healthy participants performed the task under placebo and a dopamine precursor (L-DOPA). Using ^18^F-DOPA-PET, dopamine availability in the striatum was examined at baseline to investigate its relationship to the RT distractor effect and to the L-DOPA-induced change of the RT distractor effect.

**Results:**

There was a pronounced RT distractor effect in the placebo session that increased under L-DOPA. Neither the RT distractor effect in the placebo session nor the magnitude of its L-DOPA-induced increase were related to baseline striatal dopamine.

**Conclusions:**

L-DOPA administration shifted the stability-flexibility balance towards attentional capture by distractors, suggesting causal involvement of dopamine. This finding is consistent with current theories of prefrontal cortex dopamine function. Current data can neither confirm nor falsify the inverted U-shaped function hypothesis with regard to cognitive control.

**Supplementary Information:**

The online version contains supplementary material available at 10.1007/s00213-022-06077-w.

## Introduction

Cognitive control describes the ability to allocate mental resources on behalf of goal-directed behavior (Posner and Snyder 1975; Goschke 2003, 2013; Mackie et al. 2013). In most everyday situations, this ability yields a *meta-control dilemma*, requiring a context-sensitive balancing of two antagonistic modes of attention: stability and flexibility (Goschke 2003, 2013; Goschke and Bolte 2014; Dreisbach and Fröber 2019). Stability refers to goal-directed thought, feeling, or action (e.g., focusing on a manuscript). Flexibility refers to the complementary ability to adapt to an ever-changing environment (e.g., setting aside the manuscript when smelling smoke). The balance between the two modes is thought to be regulated by several meta-control parameters such as (i) goal maintenance versus updating, (ii) goal shielding versus switching, and (iii) attentional inhibition (or interference control) versus distractibility (or interference susceptibility). These meta-control parameters are in turn assumed to be maintained by several neurotransmitters such as dopamine, noradrenaline, and acetylcholine (Noudoost and Moore 2011a; Chandler et al. 2014; Cools 2019). Despite the considerable interest in the stability-flexibility balance and its neurobiological underpinnings, experimental results from animal studies (Noudoost and Moore 2011b; Shalev et al. 2019) have not been adequately put to the test in human studies using a psychopharmacological challenge and an in vivo assessment of neurotransmission. The current study was designed to test the effect of Levodopa (L-DOPA; a precursor of dopamine) on a behavioral marker of distractibility, that is, the balance between goal-directed stimulus selection and stimulus-driven attention capture in a visual selective attention task. The study also aimed to clarify the precise nature of the relationship between dopamine and distractibility by examining (a) task performance (i.e., behavioral indicator of distractibility) in relation to individual brain dopamine availability and (b) the magnitude of the L-DOPA-induced modulation of task performance in relation to brain dopamine availability.

Neuroscience research suggests that the stability-flexibility balance is maintained by the neurotransmitter dopamine via the prefrontal cortex (PFC). According to the dual-state theory of PFC function, a relatively stronger activation of dopamine D1 receptors in the PFC is thought to uphold a “closed state” that is goal-directed and resistant to distraction by background stimuli while a D2-dominated “open state” is associated with increased cognitive flexibility (Durstewitz et al. 2000; Durstewitz and Seamans 2002, 2008; Zink et al. 2019). Past literature also suggests that dopamine signaling in the striatum has a modulating function within this process (Mier et al. 2010; Frank and Fossella 2011; Clark and Noudoost 2014; Moore and Zirnsak 2017; Cools 2019; Ott and Nieder 2019). The stability-flexibility balance can be captured within several cognitive domains, of which visual attention is one. Visual selective attention is “the selective processing of some visual stimuli (targets) in favor of others (distractors), according to their component features, identity, location within visual space or physical salience” (e.g., Noudoost and Moore 2011a). Visual search is a central aspect of visual selective attention. Specific processes within visual search can be studied with feature search, conjunction search, and spatial configuration search tasks (Müller et al. 1995; Wolfe et al. 2010; Moran et al. 2016; Petilli et al. 2020). This study addresses the influence of dopamine on the balance between focused attention (i.e., attentional inhibition/interference control) and background observation (i.e., attentional capture/distractibility/interference susceptibility) in human visual attention by means of a visual feature search task, L-DOPA administration and striatal 6-[^18^F]fluoro-L-3,4-dihydroxyphenylalanine (^18^F-DOPA) PET.

Evidence for a key role of dopamine in human visual attention mainly stems from neurocognitive-genetic approaches. For example, significant associations have been demonstrated between behavioral performance in visual attention tasks and polymorphisms in genes encoding catecholaminergic enzymes (COMT, DBH) (Shalev et al. 2019) and dopamine transporters (DAT1, SLC6A3) (Newman et al. 2014). For example, COMT Val/Val carriers, who are thought to have comparatively high levels of dopamine, showed the lowest perceptual threshold in a visual attention task (i.e., minimum exposure duration to evoke conscious perception) (Shalev et al. 2019). Further evidence comes from visual attention deficits in neuropsychiatric disorders known to involve dopamine alterations in the brain, such as Parkinson’s disease (Tommasi et al. 2015; McCoy et al. 2020) and schizophrenia (Braver et al. 1999; Keedy et al. 2009). Next to the neurotransmitter level, there is evidence in humans at the neuroanatomical level for a crucial role of the PFC and a modulating function of the striatum from neurostimulation studies (Adams et al. 2019; Wang et al. 2020), lesion studies (Voytek and Knight 2010; Wolf et al. 2014), and functional MRI studies (Anderson et al. 2007; Parhizi et al. 2018; Wang et al. 2020).

In light of the given evidence, it is reasonable to assume that the neurotransmitter dopamine and the prefrontal-striatal brain circuit together form a neural system that governs the stability-flexibility balance in visual selective attention (Braver and Cohen 2000). However, this hypothesis has not been fully put to the test in human studies, for example, by a dopaminergic intervention accompanied by neuroanatomical delineation. That is, only a few studies in humans aimed to modulate behavioral performance in a visual attention task by a pharmacological challenge of the dopaminergic system. For example, Bloemendaal et al. (2015) showed that intake of the dopamine D2 receptor agonist bromocriptine resulted in altered distractibility in a visual match-to-sample task. In their study, the distractor effect for faces (versus scenes) increased under bromocriptine compared to placebo. This effect was associated with changes in PFC connectivity. Further evidence for the involvement of dopamine and the prefrontal-striatal neurocircuit mainly comes from experimental animal studies in rodents (Chudasama and Robbins 2004b, a; Pezze et al. 2007; Agnoli and Carli 2011) and monkeys (Wardak et al. 2010; Noudoost and Moore 2011b; Cosman et al. 2018). These studies show, among a number of findings, that injections of D1/D2 receptor agonists/antagonists into the PFC and striatum modulate behavioral measures of visual (selective) attention, visual discrimination, and visual distraction. In summary, there are many indications, but limited evidence, for a causal role of prefrontal-striatal dopamine signaling in the stability-flexibility balance in humans with respect to visual selective attention.

Furthermore, there are elaborate theoretical models that predict a U-shaped relationship between brain dopamine and cognitive control (Cools and D’Esposito 2011; Papenberg et al. 2020). In line with the dual-state theory of PFC dopamine function (Durstewitz and Seamans 2008), the “inverted U-shaped” function hypothesis assumes that intermediate levels of brain dopamine stabilize a D1-dominated “closed state,” whereas both low and high levels of dopamine promote a D2-dominated “open state” (Trantham-Davidson et al. 2004; Cools 2019). With respect to a U-shaped relation, an increase in dopamine in individuals with optimal or high tonic dopamine levels should result in increased cognitive flexibility (i.e., higher distractibility) via an “open state.” In contrast, increase in dopamine in individuals with low tonic dopamine levels should result in improved goal-directed behavior via a “closed state.” In addition, there are a range of studies that also suggest a linear relationship of brain dopamine and behavior. For example, Shalev et al. (2019) reported a U-shaped association between visual sustained attention and dopamine signaling in different DBH genotypes. Yet, they also revealed a linear association between the visual perceptual threshold and the aforementioned polymorphisms in COMT. Notably, non-linear and linear mechanisms are not regarded as conflicting but as equivalent, since the nature of the relationship appears to differ among different brain regions and cognitive domains (Robbins and Arnsten 2009). In that regard, the aim of the current study was to confirm a U-shaped relationship versus a linear relationship between brain dopamine and distractibility in visual attention.

To our knowledge, no study has yet combined a dopaminergic challenge with an in vivo assessment of the individual baseline brain dopamine levels in humans to test the nature of the dopamine to behavior relationship in visual selective attention. Positron emission tomography (PET) is currently the best available in vivo approach in humans to do so. PET parameters for dopamine uptake, turnover, and washout have proven to be valuable for that purpose (Kumakura et al. 2007; Kumakura and Cumming 2009; Matsubara et al. 2011). Moreover, past studies reported associations between dopamine synthesis capacity and cognitive performance related to prefrontal-striatal circuits, such as in model-based decision-making (Deserno et al. 2015); in the stroop, trail-making, and continuous performance test (Vernaleken et al. 2007); and in working memory capacity (Cools et al. 2008; Landau et al. 2009). The study design at hand therefore included PET assessment to inform current neuroscientific models on the complex relationship between dopamine, prefrontal-striatal function, and cognitive control in humans.

**Fig. 1:**
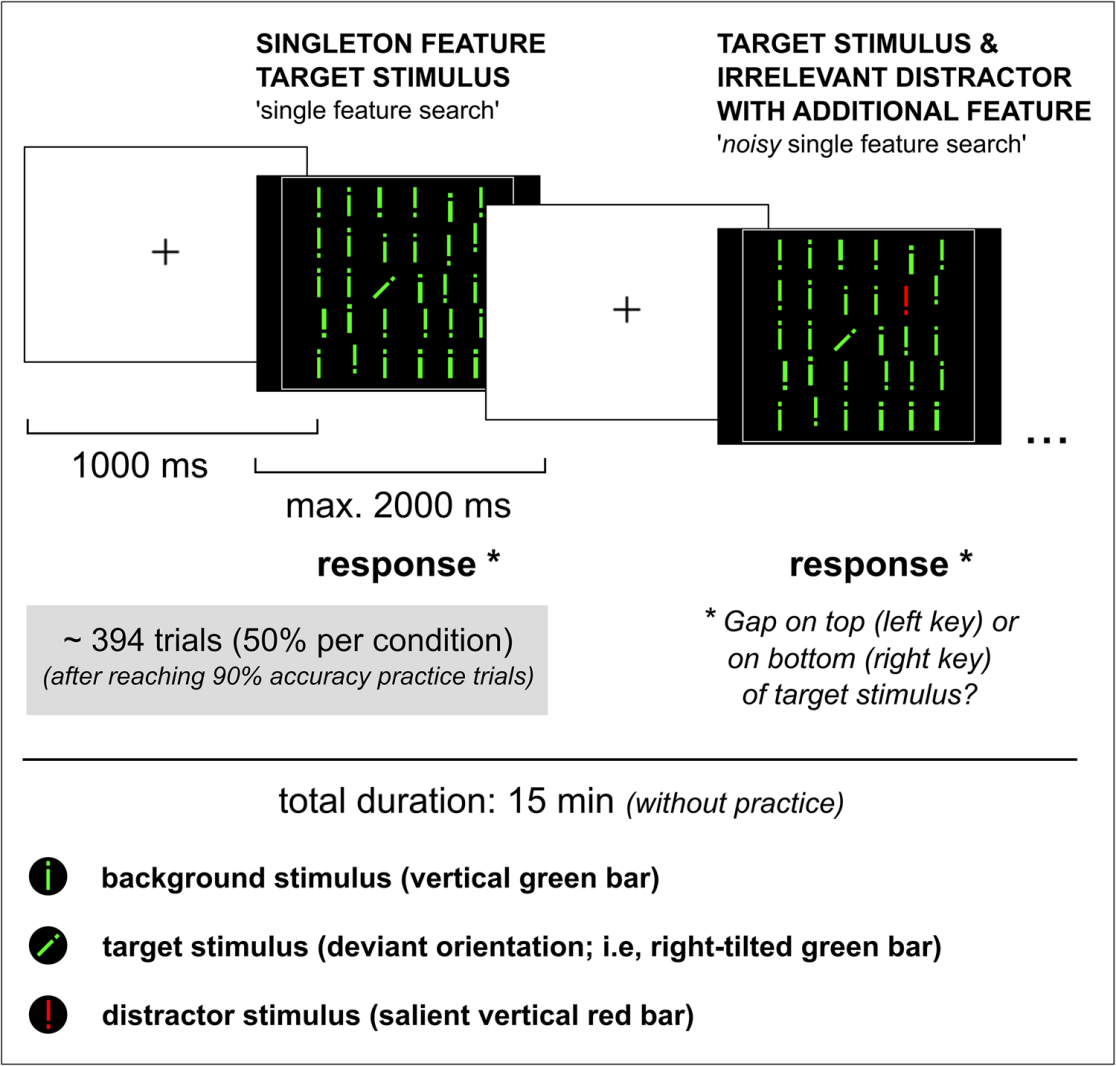
Task design. The visual search task (VST) was implemented as described in detail in the “Materials and methods” section. The figure shows an example of a target trial (left) and a distractor trial (right). Participants were asked to always indicate if the gap was on the top (left key) or on the bottom (right key) of the target stimulus

In this study, we examined the pharmacological effect of L-DOPA on a behavioral marker of distractibility (i.e., one meta-control parameter of the stability-flexibility balance) in 65 human individuals (49 males, 16 females; mean age = 36.2). We used a visual singleton feature search task (Theeuwes 1992; Müller et al. 1995; Liesefeld et al. 2017; Cosman et al. 2018). Participants were asked to identify one target stimulus that was defined by a deviant orientation (i.e., a right-tilted green bar) among homogeneous non-targets (i.e., vertical green bars) and to indicate if a gap was on the top or on the bottom of the target stimulus (see Fig. 1). About half of the trials in the visual search task (VST) included not only the singleton feature target stimulus but also a to-be-ignored salient distractor that differed from the context stimuli with respect to an irrelevant but salient (color) dimension (i.e., a red vertical bar). This task design has produced robust distractor effects in terms of increased reaction time (RT) or reduced accuracy in the distractor trials compared to target trials in the past (Theeuwes 1992; Notebaert et al. 2011; Cosman et al. 2018). In a randomized, placebo-controlled, double-blind, crossover design participants received either L-DOPA (225 mg in two doses) or placebo. L-DOPA is an amino acid precursor of dopamine that (after conversion) stimulates dopamine D1 and D2 receptors in a dose-dependent manner. To investigate the relationship (a) between the distractor effect (i.e., behavioral indicator of distractibility) and individual baseline brain dopamine levels and (b) between the magnitude of the L-DOPA-induced modulation of the distractor effect and individual baseline brain dopamine levels, ^18^F-DOPA PET was performed. Regions of interest (ROIs) in the ventral (nucleus accumbens) and dorsal striatum (caudate nucleus and putamen) were specified. Critically, prefrontal ROIs were not examined, as the ^18^F-DOPA signal in the PFC is likely to be highly susceptible to measurement error and therefore results are not reliable (Cropley et al. 2008). In each ROI, the ^18^F-DOPA parameters influx rate constant (*k*_occ_), effective distribution volume ratio (EDVR), and washout rate (*k*_loss_) were assessed.

**Fig. 2:**
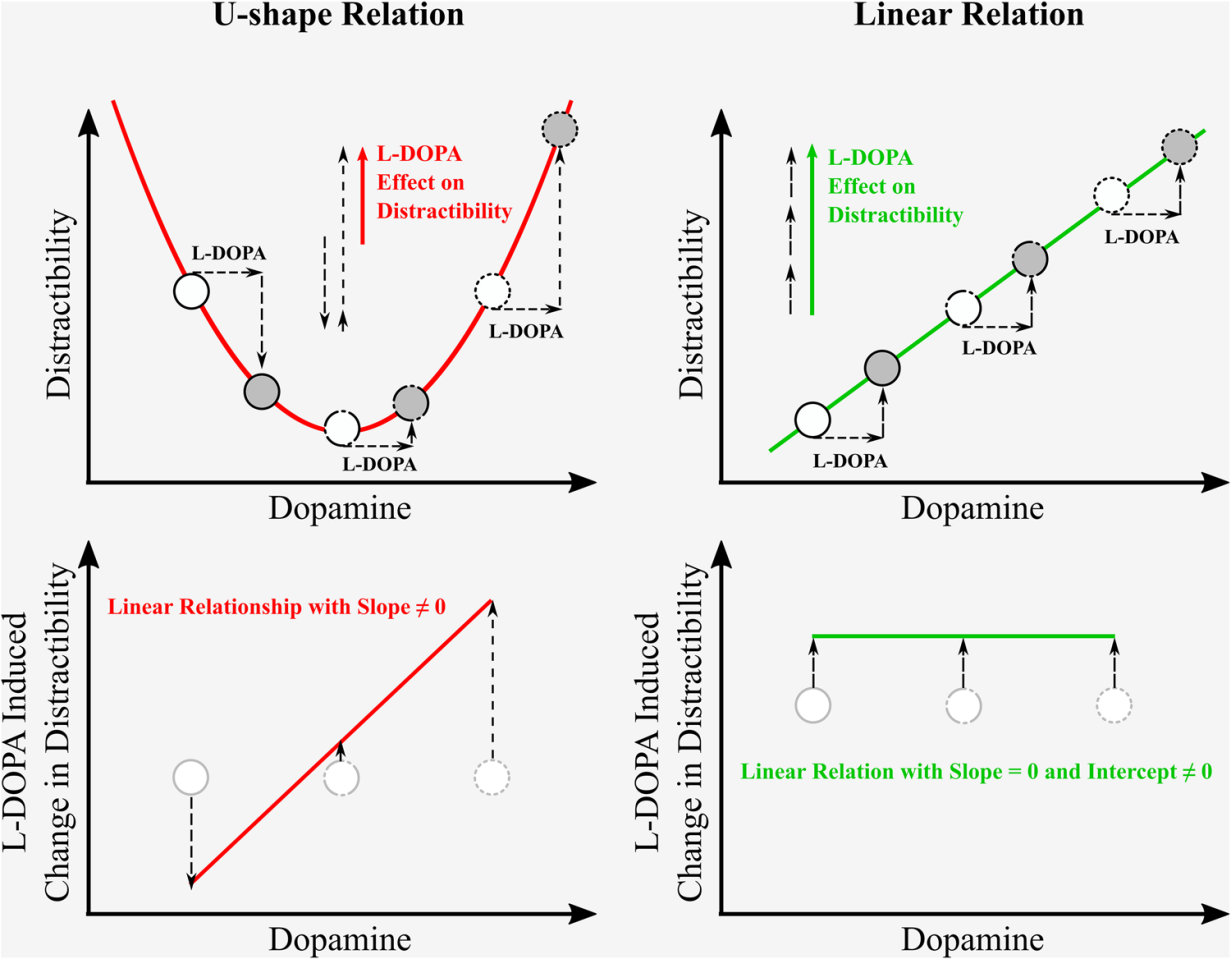
Neurobiological models. The magnitude of distractibility in the placebo session may be related to brain dopamine levels in either a U-shaped or linear fashion (upper panels). Accordingly, the magnitude of the L-DOPA-induced increase in distractibility will be either linearly related or not related to brain dopamine (lower panels)

We hypothesized that after L-DOPA intake, the stability-flexibility balance would shift toward attentional capture by distractors (i.e., increased RT distractor effect). This hypothesis rested on the assumption that dopamine levels were most likely distributed around an “optimum” in most of the healthy young adults, and excess dopamine would promote a D2-dominated “open-state” in the PFC (i.e., via a right-shift on the regression line as depicted in the upper panels of Fig. 2). Additionally, we expected one of two associations between the ^18^F-DOPA PET parameters and the magnitude of the RT distractor effect in the placebo session: (a) a U-shaped relation, that is, greater distractibility in participants with comparatively low or high baseline striatal dopamine (Fig. 2, upper left panel) or (b) a linear relation, that is, stronger distractibility with increased striatal dopamine (Fig. 2, upper right panel). Similarly, we expected either a stronger increase in distractibility in participants with comparatively high baseline striatal dopamine (Fig. 2, lower left panel) or no difference in the L-DOPA-induced behavioral modulation between participants (Fig. 2, lower right panel).

## Materials and methods

This study was part of the larger research project “Dopaminergic Modulation of Meta-Control Parameters and the Stability-Flexibility Balance” within the Collaborative Research Center 940 “Volition and Cognitive Control: Mechanisms, Modulators and Dysfunctions” (www.sfb940.de). The project aimed to investigate the effects of a dopaminergic challenge on the balance between behavioral flexibility and stability with respect to three different domains: (i) updating versus goal shielding, (ii) goal-directed versus habitual instrumental responding, and (iii) background-monitoring versus goal-directed attention. The latter domain is addressed in the study at hand. For a detailed and complete outline of the study procedures and flow of participants, we would like to refer to earlier publications (Lee et al. 2018; Kroemer et al. 2019; Petzold et al. 2019b, a).

All study procedures were approved by the Ethics Committee of the Technische Universität Dresden (TUD; EK 44,022,012) and by the German Federal Office for Radiation Protection (Bundesamt für Strahlenschutz). The experimental protocol was performed in accordance with relevant guidelines and regulations. All participants provided written informed consent and were invited to three or four study visits in total: (1) a baseline visit; (2–3) two fMRI visits including the VST, which was performed outside the MRI scanner; (4) a PET visit for a subset of participants. Based on attendance hours, participants received compensation of approximately 100 €. Data was collected from February 2014 to February 2016 at the Neuroimaging Center of the TUD and the PET Center of the Department of Nuclear Medicine at the TUD.

### Procedure

The work at hand reports on a visual singleton feature search task (VST) used to study dopaminergic modulation of the stability-flexibility balance in cognitive control. The VST was performed on each of the two fMRI visits, which were 13 days apart on average (standard deviation (*SD*) = 9). Each participant completed the task under L-DOPA and placebo (see “L-DOPA administration and measurement” below). The VST was positioned towards the end of the 5-h fMRI visits. Both fMRI visits included task-based fMRI (two-stage sequential choice task (Daw et al. 2011), set switching task (Neukam et al., in preparation)), resting-state fMRI, a value-based decision-making test battery (Pooseh et al. 2017), interviews, and blood sampling. Working memory assessment (Lewandowsky et al. 2010) and questionnaires (BDI-II (Beck et al. 1996; Whisman and Richardson 2015), STAI (Spielberger et al. 1970; Kendall et al. 1976), PSS (Cohen et al. 1983), PSQI (Buysse et al. 1989)) were additionally performed on one of the two fMRI visits, but always before L-DOPA/placebo administration. A subset of participants who completed the baseline and fMRI visits successfully was invited to the PET-Center (Department of Nuclear Medicine, TUD) on a separate day.

### Participants

The flow of participants was described in past publications (Lee et al. 2018; Kroemer et al. 2019; Petzold et al. 2019a) and is briefly summarized here. Interested members of a representative population sample stratified by age and sex (*N* = 15,778) were screened. Participants included in the study (1) were 30 to 40 years of age, (2) had no history of neuropsychiatric disorders according to the Screening Version of the Structured Clinical Interview for DSM-IV (Wittchen et al. 1997), and (3) reported no contraindications for MRI, PET, and L-DOPA administration. All participants showed proficiency in the German language. The majority of participants (about 93%) reported being right-handed. Alcohol consumption was excluded via a breath-alcohol analysis on both fMRI visits (Alcotest 6510, Drägerwerk AG & Co. KGaA, Lübeck, Germany). Intake of illicit drugs was ruled out via a urine test on the first fMRI visit (Kombi/DOA10-Schnelltest, MAHSAN Diagnostika GmbH, Reinbek, Germany). Nicotine use did not preclude study participation.

Eighty-nine participants completed the VST in both fMRI visits. Twenty-four of these participants had to be excluded (see supplement). Sixty-five participants were eligible for VST data analysis (gender: 49 males, 16 females; age at first VST: mean (*M*) = 36.2, *SD* = 3.7). Of these 65 participants, a subset of 43 participants received a PET scan and a subset of 49 participants had serum dopamine levels analyzed.

### Visual search task

#### Design

The VST had a fixed duration of 15 min with a group average of 394 trials for each visit (Fig. 1). Each trial consisted of a fixation cross, presented for 1000 ms, and a search display shown on the screen until response or for a maximum of 2000 ms. The search display consisted of a matrix of 6 × 6 vertical green bars (i.e., non-targets) each having a gap either on top or on the bottom (see Fig. 1). The target stimulus was defined by a deviant orientation (i.e., right-tilted green bar). Participants were asked to indicate if the gap was on the top (left key press) or on the bottom (right key press) of the target stimulus. About half of the trials contained not only a singleton feature target stimulus but also a to-be-ignored distractor. The distractor stimulus was defined as a red vertical bar, that is, it differed from the context stimuli with respect to an irrelevant (color) dimension. The gap in the distractor was either at the same position as in the target stimulus (compatible) or at the opposite position (incompatible). The location of targets and distractors was random, and trials with and without distractor were presented in randomized order. In both visits, participants performed a practice session until reaching an accuracy of 90% across all trial types. The mean number of practice trials for each visit was 28 (*SD* = 54, min = 10, max = 400). The VST was implemented using E-Prime presentation software, Version 2.0 (Psychology Software Tools, Sharpsburg, PA, USA). Participants were asked to carry out the task with their dominant hand.

#### Computation of outcome measures

Reaction time (RT) and accuracy were recorded. Each participant that was included for analysis reached more than 50% accuracy per trial type (target/distractor) and drug (L-DOPA/placebo). Responses with RTs greater than 2000 ms, and misses were recorded as error trials. Error trials were excluded before calculating mean RTs. Trials with an RT smaller than 200 ms were excluded from all further analyses. Within-subject trimming of RTs (e.g., by excluding trials with RTs that deviate from the individual median by more than a specified threshold; see also Wolff et al. 2016) was not performed, nor was trimming with respect to the group mean. Mean RTs and mean accuracy were calculated for each participant per trial type and drug. RTs and accuracy were additionally combined into inverse efficiency scores (IES = RT/accuracy) (Townsend and Ashby 1983; Bruyer and Brysbaert 2011) as a speed–accuracy trade-off may be balanced differently by individuals (Heitz 2014; Bogacz 2015). For example, some participants respond more slowly in favor of improved accuracy. This between-subject RT difference, which is neither due to the task nor the intervention, is taken into account by calculating IES. If IES show the same pattern of results as RTs, the results cannot be explained by differences or changes in the speed–accuracy trade-off. Interference scores (i.e., distractor effects) were calculated by subtracting RT, accuracy, and IES scores in target trials from distractor trials. Calculation of outcome measures was performed using R version 3.6.2 (R Core Team 2017).

### Working memory battery

Working memory capacity was examined at baseline and included as a covariate of no interest in the statistical analysis to control for PFC-related inter-individual differences (Lewandowsky et al. 2010; Bahmani et al. 2019; Lorenc et al. 2021). Working memory capacity was examined on the second fMRI visit before L-DOPA administration. For this purpose, the working memory task battery by Lewandowsky et al. (2010) was used with minor modifications (see supplement). The three working memory tasks used included memory updating (MU), operation span (OS), and spatial short-term memory (SSTM). A composite score (i.e., the sum of the z-standardized performance scores from three tests) was used for statistical analyses (see supplement). The task battery was implemented using Psychophysics Toolbox version 3 (Brainard 1997; Kleiner et al. 2007) within MATLAB R2010a software (The Mathworks, Inc., MA, USA).

### L-DOPA administration and measurement

**Fig. 3:**
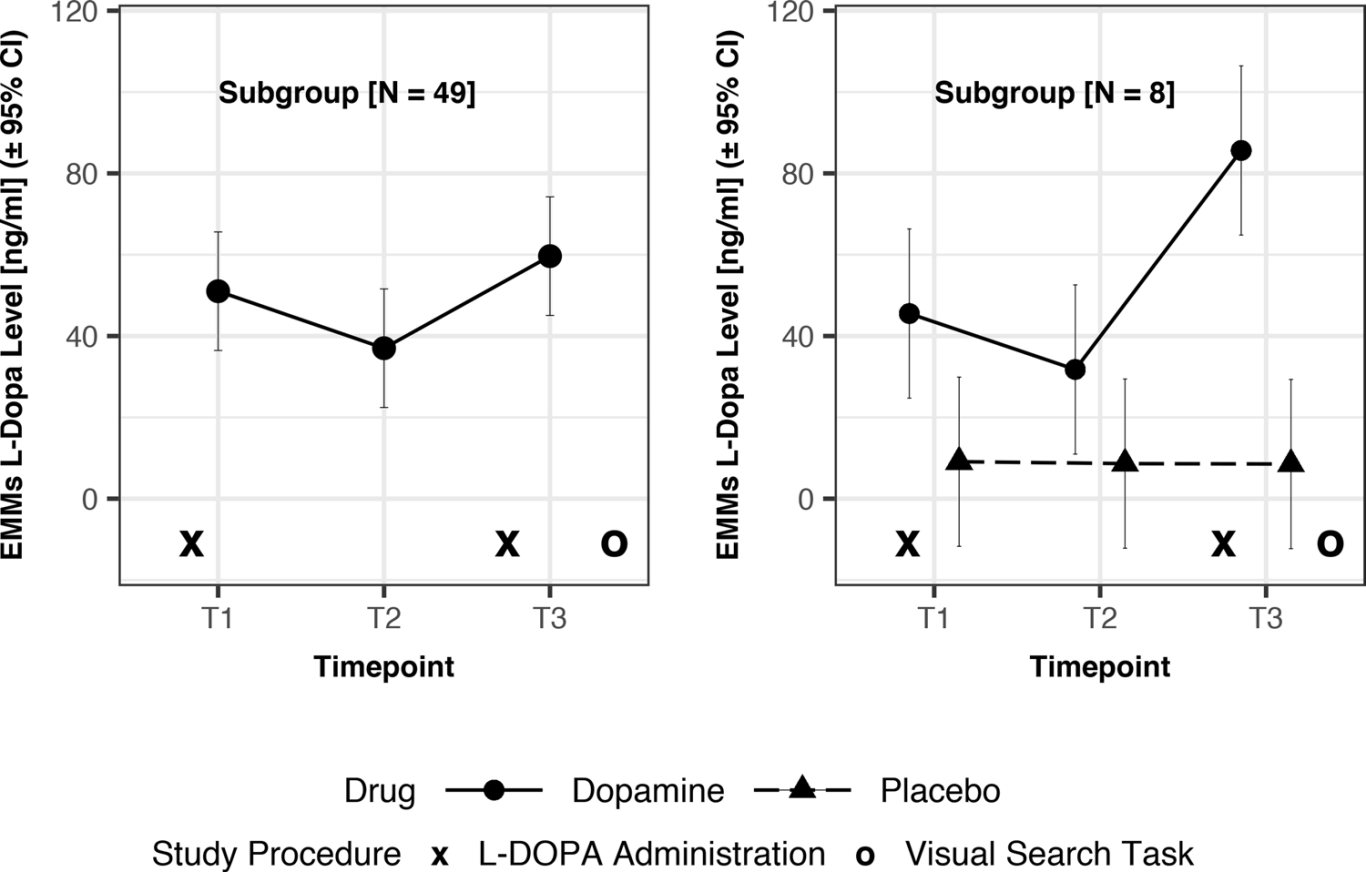
L-DOPA serum levels across time. Left panel: Presented are the estimated marginal means (EMM) for the L-DOPA serum levels in the L-DOPA session for a subsample of participants in which blood samples were available (*N* = 49). Levels are presented in chronological order from timepoint T1 (30 min after initial dose of 150 mg L-DOPA; see first “X”) to T3 (40 min after booster dose of 75 mg L-DOPA; see second “X”). The visual search task (see “O”) was performed about 75 to 90 min after the L-DOPA booster dose. Right panel: Presented are the EMMs for the L-DOPA serum levels in the L-DOPA session next to those in the placebo session for a small subsample of participants in which blood samples from both sessions were analyzed (*N* = 8)

The project used a randomized, placebo-controlled, double-blind, crossover design. Madopar (150 mg L-DOPA + 37.5 mg benserazide, a peripherally acting DOPA decarboxylase inhibitor; Roche, Grenzach-Wyhlen, Germany) or a matched placebo was administered in tablet form about 80 min after the begin of each fMRI visit. A booster dose of Madopar (75 mg L-DOPA + 18.75 mg benserazide) or a matched placebo was administered 100 min after the first dose and 75 min prior to the VST (see Fig. 3). Each participant performed the VST under both L-DOPA and placebo in separate visits. Thirty-one participants received L-DOPA at the first visit and thirty-four at the second visit.

L-DOPA serum levels during the verum session were measured (see supplement for details) for a subset of 49 participants. Serum levels during three timepoints (T1 to T3) were used to descriptively assess the course of drug exposure during the entire experimental session (Fig. 3, left panel). L-DOPA serum levels were also measured for both the verum and the placebo session in a small subsample of 8 participants to descriptively assess sufficient drug uptake (Fig. 3, right panel).

### PET

#### Acquisition

Detailed information is provided in Lee et al. (2018) and briefly summarized here. PET images were acquired with an Ingenuity TF PET/MR scanner (3 T; Philips Healthcare, OH, USA). Participants were asked to abstain from protein-containing foods on the day of the PET visit. After the initial briefing, participants received 150 mg Carbidopa (a peripherally acting DOPA decarboxylase inhibitor; Amerigen Pharmaceuticals, Lyndhurst, NJ, USA) in tablet form. Administration of Carbidopa increases the bioavailability and cerebral uptake of ^18^F-DOPA, thereby increasing the signal-to-noise ratio (Hoffman et al. 1992). Sixty minutes after Carbidopa intake, a mean ^18^F-DOPA activity of 172 ± 8.43 (range 120–185) MBq was administered intravenously at the start of the PET scan. The PET scan followed a 4-h acquisition protocol (Sossi et al. 2002).

#### Data processing

Detailed information on PET image reconstruction, parameter estimation, and image normalization is provided in Lee et al. (2018). In brief, three measures that reflect tonic dopamine levels in the brain were assessed. First is the ^18^F-DOPA influx rate constant (*k*_occ_). This parameter describes the extent of vesicular storage of fluorodopamine (FDA, to which F-DOPA was decarboxylated in the striatum), compared with a reference region in the occipital cortex. *k*_occ_ therefore reflects the uptake of dopamine within a region. Second is the the ^18^F-DOPA washout rate (*k*_loss_). This parameter indicates the rate at which dopamine is deaminated, and both FDA and its metabolites are cleared from the brain into the plasma. *k*_loss_ therefore reflects the loss of vesicular dopamine. Third is the effective distribution volume ratio (EDVR), which is the inverse of dopamine turnover (EDVR = *k*_occ_ / *k*_loss_). EDVR reflects the amount of dopamine available at steady state for vesicular storage. PET measures were acquired as averaged within three striatal region of interests (ROIs) defined in each individual’s native space: putamen, nucleus accumbens (N_acc_), caudate. Data were averaged across hemispheres in each ROI. PET measures were not assessed within prefrontal ROIs, because the ^18^F-DOPA signal in these cortical regions is likely to be more susceptible to measurement error and therefore results are less reliable (Cropley et al. 2008).

### Statistical analyses

All statistical analyses were performed in R version 3.6.2 (R Core Team 2017). Several functions from the stats package (https://www.rdocumentation.org/packages/stats/versions/3.6.2) were employed, among others. For all statistical tests, the level of significance was defined at 5% (*α* = 0.05). Figures were created using the afex_plot() function from the afex package version 0.26–0 (https://cran.r-project.org/package=afex), the ggscatter() and ggarrange() function from the ggpubr package version 0.2.5 (https://cran.r-project.org/package=ggpubr), and the ggplot() function from the ggplot2 package version 3.2.1 (https://cran.r-project.org/package=ggplot2), among others.

First, we assessed the RT distractor effect (mean RT_Distractor-Trials_ − mean RT_Target-Trials_) in the placebo session using a paired two-sample *t*-test. Reliability of the VST was assessed as for similar interference tasks in previous studies (Wolff et al. 2016, 2020; Riedel et al. 2021). That is, internal consistency (Cronbach’s *α*) was calculated by adjusting split-half correlations between odd and even trials with the Spearman-Brown prophecy formula (Brown 1910). Cronbach’s *α* was computed for the placebo session only as we expected a modulation of the RT distractor effect by L-DOPA. Internal consistency was computed for RTs in distractor trials, RTs in target trials, and the RT distractor effect (i.e., RT interference score). Mean RTs, median RTs, and standard deviations (*SD*) per drug (L-DOPA/placebo) and trial type (target/distractor) were computed. Density plots for RT distributions in each individual were created using the gghistogram() function from the ggpubr package.

Second, we tested our hypothesis that after L-DOPA intake the stability-flexibility balance would shift toward attentional capture by distractors. We performed a 2 × 2 factorial repeated measures ANOVA with the within-subject factors drug (L-DOPA/placebo) and trial type (target/distractor) using the aov_car() function from the afex package. The working memory capacity composite (WMC) score was included as a covariate. ANOVAs were separately performed for RTs, accuracy, and IES. Results of the 2 × 2 factorial repeated measures ANOVAs were consistent for RT and IES (see supplement; Table S2). Accuracy was high across conditions and sessions (*M* = 0.97, *SD* = 0.03; see also Table S2). Therefore, main analyses were restricted to the RT distractor effect.

More complex ANOVA models did not show a significant main or interaction effect of either gender or administration order (L-DOPA first session/L-DOPA second session) on RTs (see supplement; Table S3). Therefore, these two between-subject factors were not included in the main analyses as fewer factors increase statistical power to detect the effects of interest. The administration order-by-drug interaction was considered equivalent to a main effect of session (first/second), and the results were interpreted in that regard (see supplement; Fig. S4 and Fig. S5). Follow-up exploratory analyses on inter-trial effects were performed as described in the supplement. Post hoc comparisons were performed using Welch two-sample *t*-tests via the emmeans() and the pairs() functions from the emmeans package version 1.4.4 (https://CRAN.R-project.org/package=emmeans). In addition to these analyses, we assessed the correlation between L-DOPA serum levels and the magnitude of the L-DOPA-induced change in the RT distractor effect (see supplement).

Third, we tested the association between the ^18^F-DOPA PET parameters and the magnitude of the RT distractor effect in the placebo session. To test a U-shaped relationship, we separately fitted a quadratic polynomial function to the RT distractor effect as compared to the three PET measures (*k*_occ_, EDVR, *k*_loss_) in three different ROIs (putamen, N_acc_, caudate) using the lm() function from the stats package. To test a linear relationship, we fitted linear instead of quadratic functions. In addition, we investigated a U-shaped relationship between the PET data and mean RTs on distractor trials (instead of the RT distractor effect) in the placebo session.

Fourth, we tested the association between the ^18^F-DOPA PET parameters and the magnitude of the L-DOPA-induced change in the RT distractor effect. We separately fitted a linear function to the L-DOPA-induced change in the RT distractor effect as compared to the three PET measures (*k*_occ_, EDVR, *k*_loss_) in three different ROIs (putamen, N_acc_, caudate) using the lm() function from the stats package.

## Results

### Descriptive statistics on visual search task and L-DOPA intervention

Sixty-five participants had complete VST data and received both verum and placebo. Of these 65 participants, a subset of 43 participants received a PET scan and a subset of 49 participants had serum dopamine levels analyzed in the verum session. Mean RTs, median RTs, and standard deviations (*SD*) per drug (L-DOPA/placebo) and trial type (target/distractor) are presented in the supplement (Table S1), as are density plots for RT distributions in each individual (Fig. S1). L-DOPA serum levels were as expected (i) in that levels decreased after an initial peak due to the first L-DOPA administration before increasing again after the L-DOPA booster dose (Fig. 3, left panel, *N* = 49) and (ii) in that levels were higher after L-DOPA compared to placebo administration (Fig. 3, right panel, *N* = 8).

### RT distractor effect in placebo session

The analyses presented in this paragraph were conducted as an initial sanity check of the data. As expected, we found a significant RT distractor effect (mean RT_Distractor-Trials_ − mean RT_Target-Trials_) in the placebo session (16 ms) (*t*(64) = 5.64, *p* < 0.001). Internal consistency of the VST in the placebo session was excellent for mean RTs across target trials (Cronbach’s *α* = 0.99) and distractor trials (Cronbach’s *α* = 0.99). Internal consistency was much lower for the interference score (i.e., RT distractor effect (RT_Distractor-Trials_ − RT_Target-Trials_); Cronbach’s *α* = 0.55). Note that mathematically, classical reliability of a difference score decreases with an increasing correlation between the “raw” scores (Thomas and Zumbo 2012). In the current dataset, individual mean RTs in distractor trials highly correlated with individual mean RTs in target trials (*r* = 0.97, *p* < 0.001). In addition, an attenuation of the reliability coefficient for the interference score may be due to a combined measurement error from the two trial types (congruent and incongruent presentations) (Overall and Woodward 1975; Strauss et al. 2005). Hence, poor reliability of interference scores is not uncommon and has also been described for other interference tasks (Paap et al. 2020). More importantly, poor reliability of a difference score should not affect significance testing, but rather the magnitude of the effect size in the repeated measures ANOVAs used for further analyses (Chiou and Spreng 1996; Thomas and Zumbo 2012). Basic mathematical assumptions of the ANOVA are not violated by poor reliability of a difference score. Notably, internal consistency was decreased for median RTs compared to mean RTs.

### Increase of RT distractor effect by L-DOPA

**Fig. 4:**
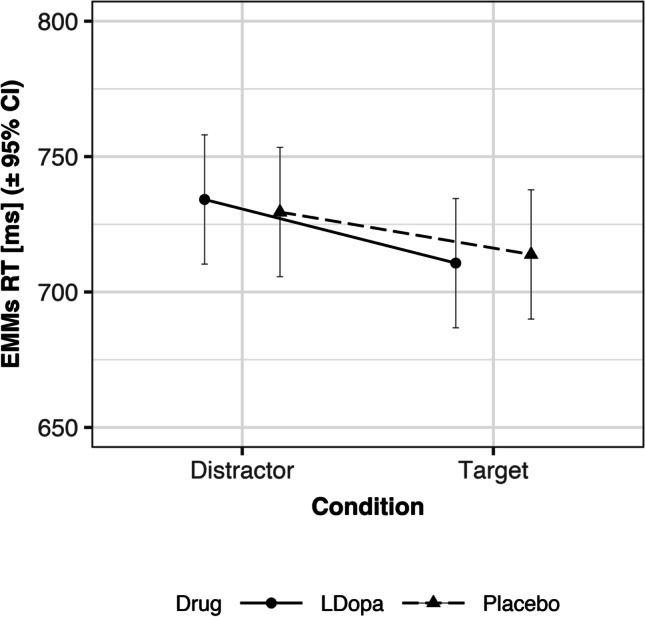
Effects of drug and trial type on reaction time (RT). Presented are the estimated marginal means (EMM) across drug (L-DOPA/placebo) and trial type (target/distractor). The RT distractor effect (RT_Distractor-Trials_ − RT_Target-Trials_; see slope) was more pronounced during the L-DOPA session compared to the placebo session

As hypothesized, L-DOPA increased the RT distractor effect. That is, the ANOVA showed a significant interaction effect between the within-subject factors drug (L-DOPA/Placebo) and trial type (target/distractor) (*F*(1,63) = 4.64, *p* = 0.035, *η*^2^_G_ = 0.0004, *η*^2^_P_ = 0.07) (Table 1). The RT distractor effect was 23 ms in the L-DOPA session (*t*(64) = 7.51, *p* < 0.001) and 16 ms in the placebo session (*t*(64) = 5.64, *p* < 0.001) (Fig. 4; Fig. S2). The effect of L-DOPA on distractor trials was opposite to target trials, numerically (see *t*-values below). However, there was no statistical difference in RTs on distractor trials between the L-DOPA and placebo session (*t*(66.4) = 0.41, *p* = 0.680). Also, there was no statistical difference in RTs on target trials between the L-DOPA and placebo session (*t*(66.4) =  − 0.29, *p* = 0.777). Follow-up exploratory analyses on inter-trial effects revealed that the L-DOPA-induced increase of the RT distractor effect was highly significant for trials that followed a distractor trial, but was not detectable for trials that followed a target trial (see supplement). The numerical differences in RTs with respect to current and previous trial type indicated adaptive attentional inhibition of distractors after distractor trials in the placebo session, whereas such adaptation did not occur in the L-DOPA session (see supplement; Fig. S8). There was no significant association between L-DOPA serum levels and the magnitude of the L-DOPA-induced increase in the RT distractor effect (see supplement; Fig. S6). Despite the effects of interest, we also observed a significant main effect of working memory capacity (*F*(1,63) = 7.80, *p* = 0.007, *η*^2^_G_ = 0.09, *η*^2^_P_ = 0.11) (Table 1), in that greater working memory capacity was associated with faster RTs (see Fig. S3). Working memory capacity, however, was not significantly modulating the RT distractor effect or its increase by L-DOPA (Table 1; Table S3).

**Table 1:** *F*-statistic: main and interaction effects of 2 × 2 factorial repeated measures ANOVA for reaction times (RT). Within-subject factors trial type (target/distractor) and drug (L-DOPA/placebo). Working memory capacity (WMC) composite score included as covariate. Values rounded to two decimals. DFn, degrees of freedom in the numerator, DFd, degrees of freedom in the denominator

	DFn	DFd	*F*	*p*	*η*^2^_G_	*η*^2^_P_
WMC composite	1	63	7.8	0.01 *	0.09	0.11
Drug	1	63	0	0.95	< 0.01	< 0.01
WMC composite × drug	1	63	0.07	0.79	< 0.01	< 0.01
Trial type	1	63	69.73	< 0.01 *	0.01	0.53
WMC composite × trial type	1	63	0.09	0.77	< 0.01	< 0.01
Trial type × drug	1	63	4.64	0.04 *	< 0.01	0.07
WMC composite × trial type × drug	1	63	1.28	0.26	< 0.01	0.02

^*^Significant; η^2^G, generalized Eta-squared; *η*^2^_P_, partial Eta-squared

### No relationship of striatal dopamine and RT distractor effect in placebo session

**Fig. 5:**
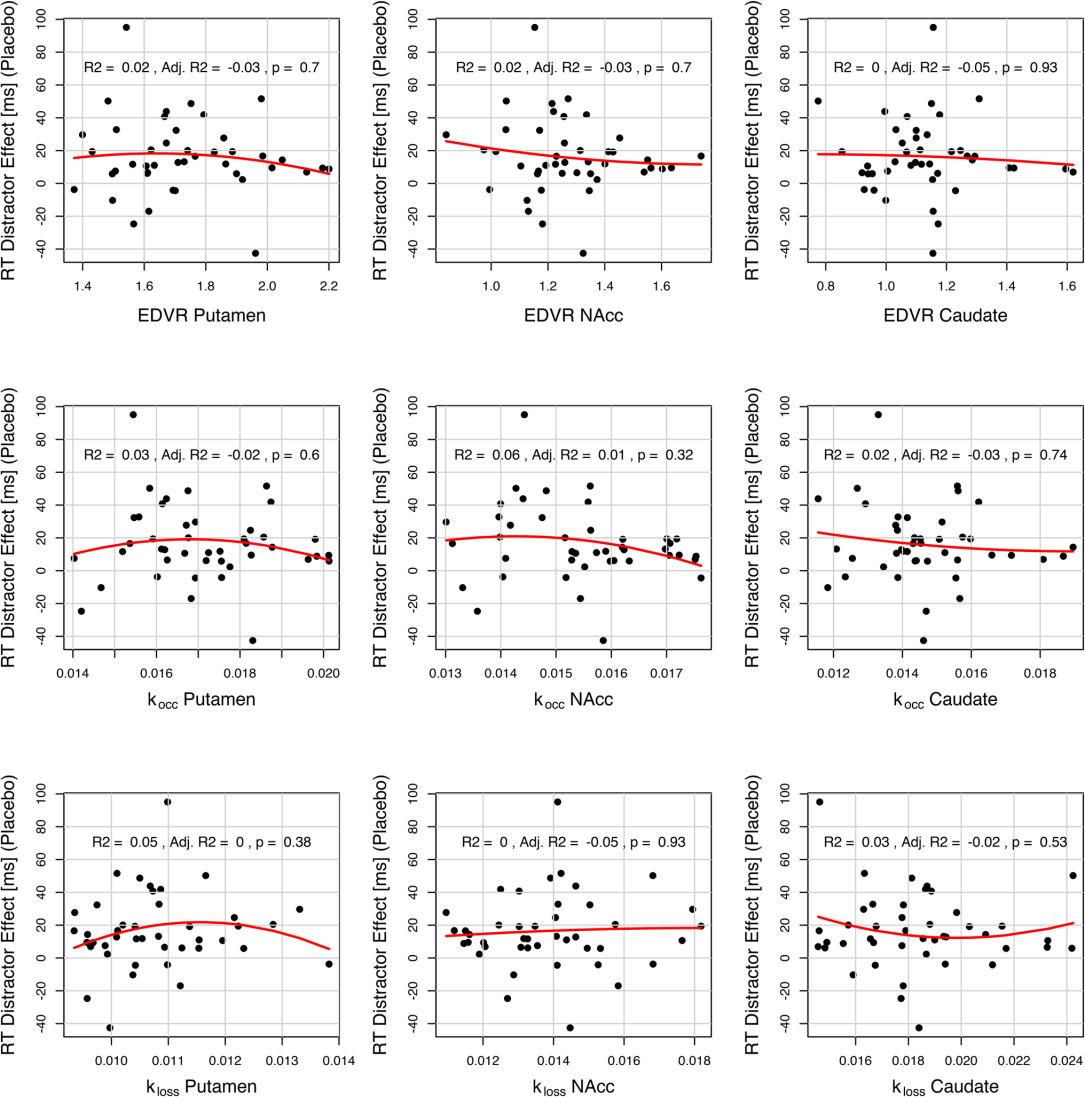
Association of RT distractor effect (placebo) and baseline striatal tonic dopamine. None of the ^18^F-DOPA-PET measures (*k*_occ_, EDVR, *k*_loss_) in any striatal ROI (putamen, N_acc_, caudate) sufficiently explained the variance in the RT distractor effect (mean RT_Distractor-Trials_ − mean RT_Target-Trials_). As presented here, there was no statistically significant quadratic model fit as expected by the “inverted U-shaped” function hypothesis. *k*_occ_, influx rate constant; EDVR, effective distribution volume ratio; *k*_loss_, washout rate; N_acc_, nucleus accumbens; RT, reaction time

**Fig. 6:**
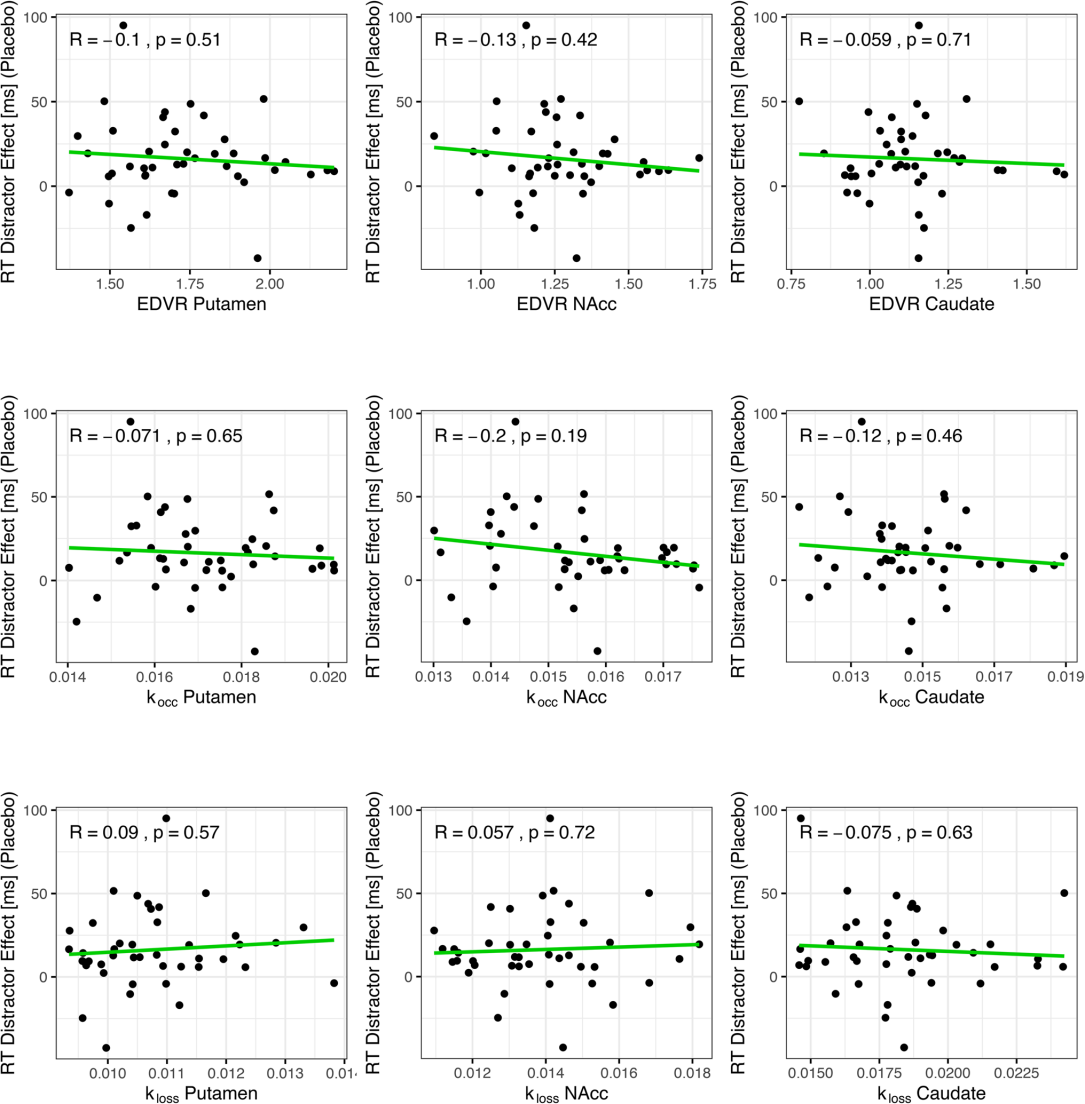
Association of the RT distractor effect (placebo) and baseline striatal tonic dopamine. None of the ^18^F-DOPA-PET measures (*k*_occ_, EDVR, *k*_loss_) in any striatal ROI (putamen, N_acc_, caudate) sufficiently explained the variance in the RT distractor effect (mean RT_Distractor-Trials_ − mean RT_Target-Trials_) in terms of a linear function. That is, there was no linear model fit that was statistically significant. *k*_occ_, influx rate constant; EDVR, effective distribution volume ratio; *k*_loss_, washout rate; N_acc_, nucleus accumbens

Contrary to our assumptions (see Fig. 2, upper panels), there was neither a quadratic nor a linear relationship between the baseline tonic dopamine level in the striatum and the RT distractor effect in the placebo session. None of the PET measures (*k*_occ_, EDVR, *k*_loss_) in any ROI (putamen, N_acc_, caudate) sufficiently explained the variance in the RT distractor effect. That is, there was neither any quadratic model fit (Fig. 5) nor any linear model fit (Fig. 6) that was statistically significant. Notably, in a supplementary analysis using RTs in distractor trials instead of the RT distractor effect as a function of striatal dopamine did suggest a quadratic relationship, especially for the ventral striatum (Fig. S9). However, none of these results would have been significant after conservative correction for multiple comparisons.

### No relationship of striatal dopamine and L-DOPA-induced increase in RT distractor effect

**Fig. 7:**
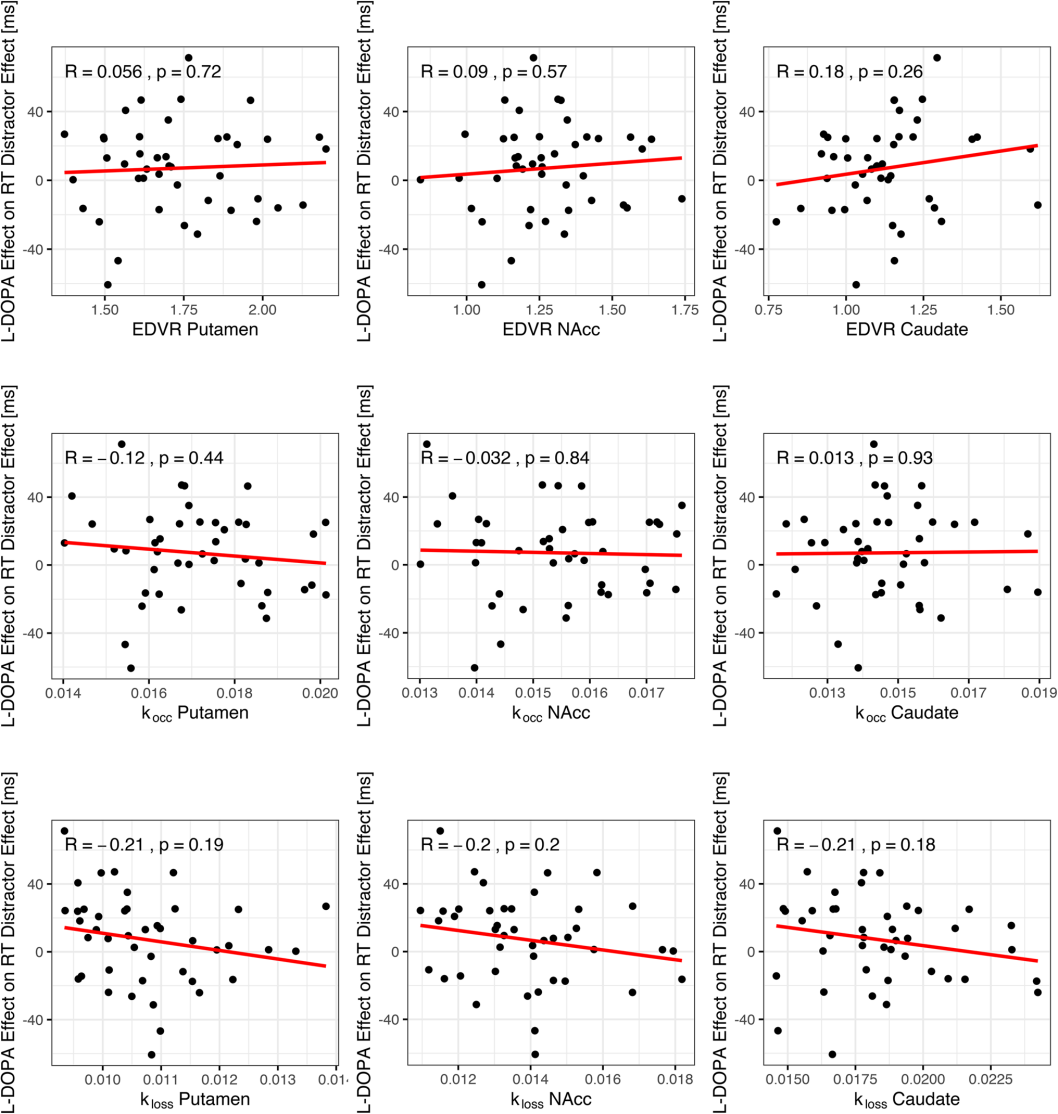
Association of the L-DOPA effect on the reaction time (RT) distractor effect and baseline striatal tonic dopamine. None of the ^18^F-DOPA-PET measures (*k*_occ_, EDVR, *k*_loss_) in any striatal ROI (putamen, N_acc_, caudate) sufficiently explained the variance in the L-DOPA effect (RT Distractor Effect _L-DOPA_ − RT Distractor Effect _Placebo_) in terms of a linear function. That is, there was no linear model fit that was statistically significant. *k*_occ_, influx rate constant; EDVR, effective distribution volume ratio; *k*_loss_, washout rate; N_acc_, nucleus accumbens

Contrary to our assumptions (see Fig. 2, lower panels), the magnitude of the dopaminergic increase of the RT distractor effect was neither governed by the baseline tonic dopamine level in the striatum nor was it constant across participants. None of the PET measures (*k*_occ_, EDVR, *k*_loss_) in any ROI (putamen, N_acc_, caudate) significantly correlated with the L-DOPA induced change in the RT distractor effect (see Fig. 7).

## Discussion

Cognitive control of visual attention requires a balance between stability (goal-orientation) and flexibility (distractibility). It has been suggested, but scarcely verified in humans, that this balance is maintained by neurotransmitters such as dopamine. Also, research indicates a U-shaped or linear relationship between dopamine availability in the brain and behavioral markers of the stability-flexibility balance. The nature of the relationship appears to differ among different brain regions and cognitive domains. Here, we used a visual search task (VST) as a behavioral marker of the stability-flexibility balance. The task was performed both after administration of the dopamine precursor L-DOPA and after placebo. PET striatal imaging is currently the best available in vivo approach in humans to assess central nervous dopamine and was employed in the current design to examine the nature of the brain dopamine to behavior relationship for visual selective attention. As hypothesized (Durstewitz and Seamans 2008), L-DOPA increased the RT distractor effect, indicating causal involvement of dopamine in the stability-flexibility balance in humans. The data could neither confirm a quadratic nor linear relationship between brain dopamine and behavior for visual selective attention (Robbins and Arnsten 2009; Cools and D’Esposito 2011). Our results call into question the extent to which a model as complex as the “inverted U-shaped” function hypothesis can be verified or refuted at all with available readouts.

Both the VST and PET assessment proved to be suitable for addressing our research questions. Behavioral VST data were consistent with past studies. We found a robust RT distractor effect in the placebo session, that is, RTs were higher in distractor trials compared to target trials (Theeuwes 1992). The magnitude of the RT distractor effect of about 16 ms in the placebo session was similar to a previous study that used the same VST (19 ms; unpublished data) and other equivalent task designs (20–40 ms in Theeuwes 1992). Our data indicated a training effect in that the RT distractor effect was slightly reduced the second time the participants performed the task (Theeuwes 1992). The PET data at hand had already shown the expected correlates with relevant parameters in other analyses, for example, with body mass index (Lee et al. 2018) and with working memory capacity (Lee et al. 2019). PET indices were within the range of different healthy adult samples at other sites (e.g., Deserno et al. 2015).

Our current analyses yielded two main results, which are presented in detail below. First, distractibility was increased by L-DOPA administration compared to placebo, as indicated by an increased RT distractor effect in the VST. Second, there was neither a U-shaped nor a linear relationship between baseline tonic dopamine in the striatum and the stability-flexibility balance as measured by the RT distractor effect. Accordingly, the magnitude of the L-DOPA-induced increase in distractibility was neither linearly related to brain dopamine levels nor constant across participants.

One approach to examining the contribution of dopamine to cognitive control is to modulate a behavioral marker of the stability-flexibility balance by means of altering dopamine availability in the brain, as pursued in this study. Given excess dopamine after L-DOPA administration in young, healthy adults, the dual-state theory of PFC dopamine function predicts a D2-dominated “open state” of the PFC that is associated with increased cognitive flexibility (i.e., increased distractibility) (Trantham-Davidson et al. 2004; Durstewitz and Seamans 2008; Cools 2019). In line with this assumption, we found that the RT distractor effect was increased by about 50% (i.e., 8 ms) in the L-DOPA session compared to the placebo session. Obviously, the statistical significance of the trial type × drug interaction on RTs was marginal and the effect size was small (especially when compared to the high inter-individual variability in RTs). However, given the low receptor and regional specificity of L-DOPA, any significant effect can be considered a successful pharmacological modulation of behavioral performance. One might critically note that L-DOPA modulated the RT interference score but had no effect on “raw” RTs in distractor trials. However, the lack of the latter effect can be explained by “a common prefrontal mechanism […] responsible for both selecting task-relevant and suppressing task-irrelevant information” (Cosman et al. 2018). Therefore, we had decided a priori to focus on the examination of the interference score as a behavioral marker of the stability-flexibility balance in human visual attention. Overall, the data at hand provide important evidence for a causal involvement of dopamine in the stability-flexibility balance in humans.

Another approach to examining the contribution of dopamine to cognitive control is to assess brain dopamine availability and its relationship to behavioral performance. The “inverted U-shaped” function hypothesis assumes optimum levels of brain dopamine for different cognitive functions (Cools and D’Esposito 2011). At the optimum level of dopamine, for example, there ought to be an optimum balance between cognitive stability and flexibility. Other data suggest that, depending on brain region and cognitive domain, there may also be a linear relationship between dopamine availability in the brain and behavioral markers of the stability-flexibility balance (Robbins and Arnsten 2009; Shalev et al. 2019). PET striatal imaging is currently the best available in vivo approach in humans to assess the nature of the brain dopamine to behavior relationship for visual selective attention. Notably, in vivo assessment of dopamine availability in humans using PET imaging is only reliably accessible for the striatum, but not for the PFC (Kumakura et al. 2007; Cropley et al. 2008).

The extended PET assessment in the current study permitted the calculation of three PET indices (instead of one) for the striatum (*k*_occ_, EDVR, *k*_loss_). A significant quadratic relationship between striatal ^18^F-DOPA-PET indices and a behavioral marker of the stability-flexibility balance would have confirmed the “inverted U-shaped” function hypothesis. A linear relationship would have confirmed a constant change in distractibility with increasing tonic dopamine in the brain. However, because dopamine effects in the PFC and striatum are sometimes thought to behave in opposite fashion, the exact direction of the relationship between PET data and behavior could not be predicted with certainty. Regardless of this matter, we found neither a quadratic nor a linear relationship in the current data. That is, participants with comparatively high or low striatal dopamine did not show a pronounced RT distractor effect. At the same time, participants’ distractibility also did not change linearly with the extend of dopamine availability.

Critically, given the between-subject design, a U-shaped and linear relationship between brain dopamine availability and the stability-flexibility balance cannot be discarded. It is entirely possible that the healthy and young participants were at their individual “optimum” dopamine levels, resulting in their individual optimal balance between target orientation and distractibility in the VST. Moreover, a U-shaped and linear relationship may be detectable only when the ratio of neurotransmitter availability and dopamine D2 receptor density is taken into account (Papenberg et al. 2020). Another explanation for the lack of a relationship is low reliability of the RT interference score, which might affect external correlates (e.g., Paap et al. 2020). With regard to the latter issue, using the “raw” RT in the distractor trials instead of the RT distractor effect suggested a quadratic relationship between brain dopamine and behavior, especially for the ventral striatum. This relationship remained even when corrected for a global measure, here working memory capacity. However, these analyses were exploratory, results were not robust, and the associations of “raw” behavioral scores cannot be interpreted as specific to cognitive control. In summary, analyses confirmed neither the “inverted U-shaped” function hypothesis nor a linear relation with regard to striatal PET imaging and distractibility in the visual search task.

Because the “inverted U-shaped” function hypothesis could neither be confirmed nor rejected using our placebo and baseline data for the reasons discussed above, a positive correlation between brain dopamine and the L-DOPA-induced increase in distractibility (Fig. 2, left panels) was not to be expected. Consequently, it was observed in our data that the magnitude of the L-DOPA-induced increase in distractibility was independent of tonic dopamine in the brain at baseline (Fig. 7). Notably, the lack of a correlation neither supports nor contradicts a U-shaped versus linear relationship for visual selective attention, because there are no corresponding associations between brain dopamine and behavior in the placebo session (see Figs. 5 and 6). More specifically, a constant L-DOPA-induced increase in the RT distractor effect could be explained in terms of both a linear relation (Fig. 2, left panels) and a U-shaped function. With regard to the latter theory, it could be that L-DOPA, assuming individually “optimum” levels of dopamine, resulted in a constant rightward shift on the U-shaped curve from the minimum in each participant. Either way, high inter-subject variability in the L-DOPA-induced increase in distractibility (see y-axis in Fig. 7) suggests an additional involvement of other neurotransmitters, such as noradrenaline and acetylcholine, in regulating executive circuits and their impact on behavioral correlates of visual selective attention (Noudoost and Moore 2011a; Chandler et al. 2014; Cools 2019).

Taken together, the current study succeeded in confirming a causal role of the neurotransmitter dopamine in the stability-flexibility balance during visual selective attention in humans. Excess dopamine shifted the balance away from goal-directed stimulus selection towards stimulus-driven attention capture (i.e., towards distractibility). The use of extended ^18^F-DOPA PET at baseline in a large participant sample did not provide further insight into the neuronal mechanisms underlying this effect. However, our PET results challenge the “inverted U-shaped” function hypothesis by calling into question the extent to which this complex model can be verified or refuted at all with available readouts.

The L-DOPA effect of increased distractibility in the visual search task can be explained with regard to three theoretical models, although direct evidence from neuroimaging is lacking. First, the effect is in line with the dual-state theory of PFC dopamine function (Durstewitz and Seamans 2008). That is, an “open state” might have been induced via increased D2 receptor activation in the PFC as a result of excess dopamine availability. Follow-up exploratory analyses on inter-trial effects provided a more informed view in this regard (see supplement for details). The results indicated that L-DOPA inhibited the upregulation of attentional inhibition in response to distracting stimuli, leading in particular to increased distractibility after previous distractor trials. This finding can be interpreted as reduced adaptive top-down control of visual selective attention as a result of increased brain dopamine levels (i.e., an “open state” of the PFC). Nevertheless, other explanations are equally important. Second, gating of information to the PFC via the striatum might have been modulated by L-DOPA, in that excess dopamine amplified (distracting) visual input to prefrontal cortex from sensory regions in the posterior cortex (Donnell and Grace 1995; Horvitz 2002; Murer and O’Donnell 2016). Third, the interplay between the different actions of dopamine at different prefrontal and striatal sites and the resulting equilibrium state of the neuronal system (Cools 2019) may have been imbalanced towards flexibility in a more complex manner by increasing dopamine availability in all neuronal pathways in terms of the unspecific dopaminergic challenge.

Despite the demonstrated effect of L-DOPA on the stability-flexibility balance, we would like to discuss below the potential impact of specific task characteristics on our main finding. Although the RT distractor effect of 16 ms in our visual search task was within the range of previous studies, RT differences between distractor and target trials of significantly greater magnitude were obtained under certain experimental conditions in other studies. First, a low frequency of distractor trials (down to 20%) goes along with a much larger RT distractor effect (up to 200 ms) compared with moderate (50%, as used in the current study) or high frequency (up to 80%) (Müller et al. 2009; Won et al. 2019). Second, a distractor in the same dimension as the target (e.g., distractor = 90° tilted and target = 30° tilted) has been shown to produce interference scores of about 80 ms compared to about 10 ms when using a color distractor (Zhang et al. 2021). Third, “pop out” of a tilted target is present down to a 3° angle (Liesefeld et al. 2016), which is much lower than in the current study. This fact suggests that our target might have been rendered too competitive for the first allocation of attention, resulting in a rather small RT distractor effect. Notably, some of these aspects were addressed in our pilot studies. We tested, for example, other target types (i.e., form of a triangle, hatching of the bar) in order to include a switch condition (not done in the current study). This manipulation had no relevant effect on the RT distractor effect. Furthermore, we assume that the choice of a color distractor did not necessarily have to result in a reduced interference score on average due to counteracting factors (i.e., high distraction at the beginning, which only decreases over the course of the task). Frankly, however, other aspects were not considered during the piloting but offer a great potential for optimization and manipulation in future studies that combine a pharmacological intervention with a visual search task. Notably, even though the aforementioned design characteristics may well be considered a caveat in the operationalization of distractibility per se, in retrospect, they may also have led to a benefit for our pharmacological challenge. Compared to a low 20% frequency of distractors the moderate 50% frequency in our task version could have driven top-down control towards a maximum and thus reduced distractibility to a minimum during the placebo session (see also Müller et al. 2009). This circumstance would then render our task even more sensitive for the detection of a postulated increase in distractibility by L-DOPA; ceiling effects would be avoided. Moreover, the moderate distractor frequency of 50% permitted a robust exploratory analysis of inter-trial effects.

Moreover, the current study certainly had several limitations that impeded the investigation of neural mechanisms and that of the “inverted U-shaped” function hypothesis in particular. These limitations include (i) the lack of neuroimaging data for both sessions, (ii) no assessment of prefrontal dopamine metabolism, (iii) no assessment of individual dopamine receptor density, (iv) low specificity of the dopaminergic intervention, and (v) a lack of separate task sessions under varying drug dose. On the one hand, future projects could address these limitations, and a stepwise approach would likely be most promising. Studies could first identify a task with a robust behavioral marker of the stability-flexibility balance that is substantially, specifically and preferably bidirectionally modulated by a pharmacological intervention. Agents such as bromocriptine (selective dopamine D2 receptor agonist), amisulpride and sulpiride (selective dopamine D2 receptor antagonists), and PDE10A inhibitors (selective modulation of dopamine receptor signaling in the striatum) have proven to be suitable candidates (Bloemendaal et al. 2015; Schülke and Brandon 2017; Hauser et al. 2019; Westbrook et al. 2020). For example, Bloemendaal et al. investigated behavioral distracter-resistance and found increased distractor cost, compared to placebo, after administration of bromocriptine, but not after administration of L-DOPA (2015). In a second step, repeated neuroimaging could be employed to examine PFC and striatal involvement under the above specific dopaminergic agents in a within-subject design. Of course, novel approaches such as functional MRI scanning along with PET assessment (Salami et al. 2019), combined PET and structural MRI examination (D’Ambrosio et al. 2021), PET assessment of multiple neurotransmitters (Voon et al. 2020), and prefrontal [^11^C]raclopride-PET (Papenberg et al. 2020) to measure receptor density, could be applied as well. To disentangle which specific sequence of the attentional mechanism dopamine is involved in, drug interventions could be implemented in an EEG design similar to that of Liesefeld et al. (2017). On the other hand, even such enormous scientific efforts might not be sufficient to prove or falsify the current complex, but rather loosely defined theoretical models, the “inverted U-shaped” function hypothesis in particular. Importantly, these models have their raison d’être, could accurately represent brain-behavior relationships in humans, and are validated in humans for some cognitive domains such as working memory. But they need further refinement to make them more accessible to scientific inquiry in other cognitive domains. This refinement might involve clear neurocomputational frameworks and may precede the conduction of further (invasive and costly) studies in humans.

Although the magnitude of the L-DOPA-induced modification of the RT distractor effect is of little practical utility in terms of pharmacological treatment, our findings are relevant to understanding cognitive impairment and cognitive control dysfunction in a wide range of neuropsychiatric disorders (Goschke 2014). Prefrontal-striatal neurocircuits and dopamine are, for example, critical in the development, neurobiology, and clinical presentation of schizophrenia (Simpson et al. 2010; Dandash et al. 2017; Waltz 2017; Arnsten et al. 2017; McCutcheon et al. 2019; Heinz et al. 2019). Moreover, attentional deficits are particularly prominent within schizophrenia compared to other cognitive domains and in schizophrenia compared to bipolar disorder (Lee et al. 2013; Li et al. 2020), and the importance of disruption of prefrontal-striatal dopamine for attentional deficits in schizophrenia has already been implicated in animal research (Chudasama and Robbins 2004a, b). Thus, further investigation of causal relationships between prefrontal-striatal dopamine and specific aspects of attention in healthy humans is critical for subsequently understanding the pathophysiology of schizophrenia and for treatment approaches. The present study may be a first step toward gaining an understanding of specific visual search deficits in schizophrenia (Fuller et al. 2006; Gold et al. 2007; Elahipanah et al. 2010).

In conclusion, our findings highlight causal involvement of dopamine in cognitive control and in selective visual attention in particular. The fact that extensive PET assessment in a large participant sample did not yield additional insights on underlying neuronal mechanisms should encourage further refinement of theoretical neurobiological models of the stability-flexibility balance and inform future study designs. For these two reasons, our study is a key building block for future experimental psychology research into the neurobiological foundation of cognitive control. Our findings are also relevant to clinical psychiatry and psychotherapy, as they can be drawn upon for developing theories on the transdiagnostic neurocognitive mechanisms involved in pathogenesis (e.g., vulnerability factors). Ultimately, these core mechanisms may be targeted in treatment.

## Supplementary Information

Below is the link to the electronic supplementary material.

Supplementary file1 (DOCX 961 KB)
