# Supplementary material for: L-DOPA administration shifts the stability-flexibility balance towards attentional capture by distractors during a visual search task
Source: Psychopharmacology (Berl). 2022 Feb 11;239(3):867–85. doi: 10.1007/s00213-022-06077-w (PMC8891202; doi:10.1007/s00213-022-06077-w)
Supplement: Supplementary file 1 — Supplementary file1 (DOCX 961 KB) [file 213_2022_6077_MOESM1_ESM.docx]

**SUPPLEMENT**

**L-DOPA administration shifts the stability-flexibility balance towards attentional capture by distractors during a visual search task**

*Psychopharmacology*

P. Riedel ^1^, I. M. Domachowska ^2^, Y. Lee ^1^, P. T. Neukam ^1^, L. Tönges ^3^, S. C. Li ^2,4^, T. Goschke ^2, a^ & M. N. Smolka ^1, a*^

^1^ Department of Psychiatry and Psychotherapy, Technische Universität Dresden, Fetscherstraße 74, 01307 Dresden, Germany

^2^ Department of Psychology, Technische Universität Dresden, Zellescher Weg 17, 01069 Dresden, Germany

^3^ Department of Neurology, Ruhr University Bochum, St. Josef-Hospital, Gudrunstraße 56, 44791 Bochum, Germany

^4^ Centre for Tactile Internet with Human-in-the-Loop, Technische Universität Dresden, Georg-Schumman-Str. 9, 01187 Dresden, Germany

^a^ shared last authorship, equal contributions

* Correspondence: Michael N. Smolka, Section of Systems Neuroscience, Technische Universität Dresden, Würzburger Straße 35, 01187 Dresden, Germany; michael.smolka@tu-dresden.de

# Supplemental Methods

## Participants

Twenty-four participants had to be excluded. Six participants were not eligible due to specific issues such as a positive drug test, less than two hours of sleep before participation, administration of placebo twice, and difficulty understanding task instructions. One participant did not have any valid data for working memory capacity. Seventeen participants achieved 90% accuracy after practice trials, but scored well below chance level (50%) in one of the conditions (Target/Distractor) in one of the sessions and were therefore excluded.

## Working Memory Battery

Working memory capacity was examined on the second fMRI visit before L-DOPA administration. The working memory task battery by Lewandowsky et al. (2010) was implemented using Psychophysics Toolbox version 3 (Brainard 1997; Kleiner et al. 2007) within MATLAB R2010a software (The Mathworks, Inc., MA, USA). In the current study, the battery has been slightly adapted from the original version.

We used the memory updating task (MU), the operation span task (OS), and the spatial short-term memory task (﻿SSTM). The sentence span task (SS) was excluded because it was not considered relevant to the tasks of interest in the project (i.e., Two-stage Sequential Choice Task, Set Switching Task, Visual Search Task). The OS was slightly modified from the original version. For each trial, participants were presented with a series of alternating arithmetic equations and consonants (ranging between 4 to 8 pairs of equations and their following consonant per trial). When an equation (e.g. ‘3 + 3 = 5’) was presented on the screen, the participant had to press the left button if the equation was correct and the right button if it was false. At the end of the trial, participants had to type the consonants in the order they were presented. MU and SSTM were implemented as in the original version. All participants were asked to carry out the tasks with their dominant hand.

Further modifications were as follows. As in the original version, the set size order was fixed for all participants, but the stimulus presentation within tasks was randomized. In contrast to the original battery we included practice trials that were presented after a set of instructions at the beginning of each task. We used German language and a different format for data storage.

If only one of three task results (MU, OS, or SSTM) was missing, the data were imputed. Missing data were minimal (0.01%). Imputation of missing data was performed using the Amelia package in R (Honaker et al. 2015). The Expectation-Maximization (EM) algorithm was run once on the full dataset (instead of imputation based on bootstrapped data). A z-standardized performance score was computed for each task. A composite score was computed for each participant by summing the z-standardized scores of the three working memory tasks (MU, OS, SSTM). The composite score was z-standardized and used as a trait marker of working memory capacity in the statistical analyses (i.e. as a covariate in the ANOVAs).

## L-DOPA: Measurement of Serum Levels

Venous puncture was performed by medical laboratory scientists and 7.5 ml of blood were drawn and collected in EDTA-test tubes containing 0.5% sodium disulfite solution. After rapid centrifugation of the whole blood sample and after pipetting, the serum samples were immediately frozen at −80°C until further analysis. L-DOPA serum level assessment was performed at the Neurochemical Laboratory of the Katholische Klinikum (Catholic Clinic) in Bochum (Gudrunstraße 56, 44791 Bochum). Reversed-phase high performance liquid chromatography (HPLC) was performed in combination with electrochemical detection (HPLC Thermo Fisher UltiMateTM 3000 with electrochemical ECD-3000RS detector; Thermo Fisher Scientific, Waltham, MA, USA) and degaser for the measurement of levodopa levels in plasma. Plasma was diluted with a factor of 1:1.95 before assessment. Then 0.450 mL of ice-cold perchloric acid 0.7 M and 0.010 ml EDTA 0.1% were added to 1.0 mL of prediluted plasma. Next, the samples were centrifuged for 30 min with 12.000 U/min at 4° C (Hettich Centrifuge Mikro 220R; Tuttlingen, Germany) before application to the HPLC device.

Blood sampling was conducted at four timepoints: [1] Before the initial L-DOPA dose (T0), [2] 30 min after the initial L-DOPA dose (T1), [3] 90 min after the initial L-DOPA dose (T2), [4] 40 min after the L-DOPA booster dose (T3). L-DOPA serum levels were available only for a subsample of 49 participants and not for T0 because of inaccurate analytical methods used in an initial attempt to measure L-DOPA concentrations. Serum levels at timepoints T1 to T3 were used to descriptively assess the course of drug exposure during the entire experimental session (Fig. 2, left panel). L-DOPA serum levels closest to the VST (i.e., T3, 35 min before the VST) were used for bivariate correlation with the L-DOPA-induced change in the RT distractor effect (N = 49). L-DOPA serum levels were measured for both the verum and the placebo session in a small subsample of 8 participants to descriptively assess sufficient drug uptake (Fig. 2, right panel). To assess potential effects of storage duration on measured L-DOPA serum levels, we additionally correlated L-DOPA levels at T3 with the days the samples were stored in the freezer.

## Statistical Analyses: Modulation of RT Distractor Effect by L-DOPA: Effects of Gender and Dopamine Application Order

To assess potential confounding effects by gender and administration order (L-DOPA first session/L-DOPA second session) we performed a 2 X 2 X 2 X 2 mixed-design ANOVA with the above between-subject factors in addition to the within-subject factors trial-type (Target/Distractor) and drug intervention (L-DOPA/Placebo). We used the aov_car() function from the afex package. The same analysis was used to examine a session effect, which is equivalent to the administration order X drug interaction.

## Statistical Analyses: Modulation of RT Distractor Effect by L-DOPA: Inter-trial Effects

To assess inter-trial effects (i.e., whether the trial-type in the previous trial affected performance in the current trial), we performed a 2 X 2 X 2 factorial repeated measures ANOVA on RTs with the within-subject factors current trial-type (Target/Distractor), previous trial-type (Target/Distractor) and drug intervention (L-DOPA/Placebo). We used the aov_car() function from the afex package. Post-hoc comparisons were performed using Welch two-sample t-tests via the emmeans() and the pairs() functions from the emmeans package.

## Statistical Analyses: Relationship of Striatal Dopamine and mean RTs on Distractor Trials in Placebo Session

In addition to the main analyses, we investigated a U-shape relationship between the PET measures and mean RTs on distractor trials in the placebo session. As for the main analyses, we fitted a quadratic polynomial function using the lm() function. Statistical results were not corrected for multiple comparisons.

# Supplemental Results

Descriptive Statistics on Visual Search Task and L-DOPA Intervention

Sixty-five participants had complete VST data and received both verum and placebo. Mean RTs, median RTs and standard deviations (SD) per drug (L-DOPA/Placebo) and trial-type (Target/Distractor) are presented in Table S1. Density plots for RT distributions in each individual are presented in Fig. S1.

## Modulation of RT Distractor Effect by L-DOPA: Effects of Gender and Dopamine Application Order

A 2 X 2 X 2 X 2 mixed-design ANOVA with the between-subject factors gender and administration order (L-DOPA first session/L-DOPA second session) and the within-subject factors trial-type (Target/Distractor) and drug intervention (L-DOPA/Placebo) yielded no significant main or interaction effect for gender and administration order (Table S2). The significant administration order X drug interaction [F(1,60) = 25.53, p < 0.001, η²_P_ = 0.30] is equivalent to a main effect of session in the current study design (see also Fig. S4 and S5). Participants performed the task about 51 ms faster in the second compared to the first session [t(63) = -5.79, p < 0.001]. There was a trend for a smaller RT distractor effect in the second compared to the first session [F(1,60) = 3.47, p = 0.066, η²_P_ = 0.05].

L-DOPA significantly increased the RT distractor effect by about 8 ms (Fig. 4 and Fig. S2). However, there was no significant linear relationship between the L-DOPA serum level during the verum session and the magnitude of the L-DOPA-induced increase of the RT distractor effect (Fig. S6). The direction of the association, however, is in line with an expected stronger modulation with higher L-DOPA serum levels (Fig. S6). Although storage duration of the serum samples before laboratory analysis did not correlate with L-DOPA serum levels (Fig. S7), effects of storage and other aspects (blood collection, transport, etc.) on the accuracy of the L-DOPA serum levels cannot be excluded. Note that L-DOPA serum levels were actually expected to be higher after the administered doses of Madopar.

## Modulation of RT Distractor Effect by L-DOPA: Inter-trial Effects

A 2 X 2 X 2 factorial repeated measures ANOVA with the within-subject factors current trial-type (Target/Distractor), previous trial-type (Target/Distractor) and drug intervention (L-DOPA/Placebo) yielded a significant modulation of the L-DOPA effect on distractibility by previous trial-type (Table S4). Post-hoc analyses revealed that the L-DOPA induced increase of the RT distractor effect found in the main analyses was clearly present for trials that followed a distractor trial [t(64) = 3.27, p = 0.002], but was not detectable for trials that followed a target trial [t(64) = -0.03, p = 0.973] (see also Fig. S8). Numerically, participants responded about 9 ms slower on distractor trials that followed a distractor trial in the L-DOPA compared to the placebo session. At the same time, participants responded about 7 ms faster (numerically) on target trials that followed a distractor trial in the L-DOPA compared to the placebo session. This finding suggests reduced top-down control under L-DOPA. That is, an unconscious but adaptive upregulation of attentional inhibition on distractor trials to shield a distractor in the following trial is not as pronounced under L-DOPA as in the placebo session. Such reduced upregulation under increased dopamine levels in the brain results in faster responses, when a target trial follows a distractor trial, because fewer cognitive resources had to be recruited in advance. Such reduced upregulation of attentional inhibition, however, prevents better shielding of a distractor in a distractor trial that followed a distractor trial. In neither the L-DOPA session nor the placebo session did further post-hoc-testing reveal a significant RT difference either between (i) distractor trials that followed target trials and (ii) distractor trials that followed distractor trials or (iii) target trials that followed distractor trials and (iv) target trials that followed target trials (all p > 0.1). The order of the four ‘trial-types’ in terms of their mean RT was (i) – (ii) – (iii) – (iv) in the placebo session (in line with Müller et al. 2009) and (ii) – (i) – (iv) – (iii) in the L-DOPA session (see Fig. S8).

## Relationship of Striatal Dopamine and mean RTs on Distractor Trials in Placebo Session

Forty-three participants had completed the VST at both the first and second session as well as PET imaging. As described in the main manuscript, there was neither a quadratic nor a linear relationship between any PET measure in any striatal ROI and the RT distractor effect in the placebo session (Fig. 5 and Fig. 6). Further analyses using mean RTs on distractor trials in the placebo session (instead of the RT distractor effect) revealed an inverted U-shape relationship with EDVR (i.e., amount of dopamine available at steady state) and *k*_loss_ (i.e., loss of vesicular dopamine) in the ventral striatum (i.e., N_acc_) (Fig. S9; middle panels). Also, there was a trend level quadratic relationship between *k*_occ_ (i.e., uptake of dopamine) in the dorsal striatum (i.e., caudate) and the mean RTs on distractor trials in the placebo session (Fig. S8; right panels). These results did not change substantially when adjusting mean RTs on distractor trials for working memory capacity. However, none of the results would have been significant after conservative correction for multiple comparisons.

# References (5)

Brainard DH (1997) The Psychophysics Toolbox. Spat Vis 10:433–436. https://doi.org/https://doi.org/10.1163/156856897X00357

Honaker J, King G, Blackwell M (2015) Amelia II: A Program for Missing Data . J Stat Softw. https://doi.org/10.18637/jss.v045.i07

Kleiner M, Brainard D, Pelli D (2007) What’s new in Psychtoolbox-3? Perception 36:1–16

Lewandowsky S, Oberauer K, Yang LX, Ecker UK (2010) A working memory test battery for MATLAB. Behav Res Methods 42:571–585. https://doi.org/10.3758/BRM.42.2.571

Müller HJ, Geyer T, Zehetleitner M, Krummenacher J (2009) Attentional Capture by Salient Color Singleton Distractors Is Modulated by Top-Down Dimensional Set. J Exp Psychol Hum Percept Perform 35:1–16. https://doi.org/10.1037/0096-1523.35.1.1

# Table S1

*Descriptive Statistics for Reaction Time (RT).* Mean, median, standard deviation (SD), maximum (Max), and minimum (Min) for RTs are presented by Trial-type (Target/Distractor) and Drug (L-DOPA/Placebo). In addition, descriptive statistics are presented for the RT distractor effect in each drug condition.

| **Descriptive Statistics** | **RT (ms)** | | | | | |
| --- | --- | --- | --- | --- | --- | --- |
|  | **L-DOPA**  **Distractor** | **L-DOPA**  **Target** | **L-DOPA**  **Distr. Effect** | **Placebo**  **Distractor** | **Placebo**  **Target** | **Placebo**  **Distr. Effect** |
| **Sample Mean** | 734 | 711 | 23 | 730 | 714 | 16 |
| **Sample Median** | 700 | 693 | 22 | 731 | 714 | 12 |
| **Sample SD** | 117 | 109 | 25 | 93 | 94 | 22 |
| **Sample Max** | 1132 | 1047 | 106 | 1026 | 1050 | 95 |
| **Sample Min** | 551 | 541 | -28 | 544 | 539 | -43 |

# Table S2

*F-statistic: Main and interaction effects of 2 X 2 factorial repeated measures ANOVA for reaction times (RT; left), inverse efficiency scores (IES; middle), and accuracy (right).* Within-subjects factors Trial-type (Target/Distractor) and Drug (L-DOPA/Placebo). Working Memory Capacity (WMC) composite score included as covariate. Values rounded to two decimals. DFn = Degrees of Freedom in the numerator, DFd = Degrees of Freedom in the denominator, * Significant, η²_P_ = partial Eta-squared.

|  | | | **RT** | | | **IES** | | | **Accuracy** | | |
| --- | --- | --- | --- | --- | --- | --- | --- | --- | --- | --- | --- |
|  | **DFn** | **DFd** | **F** | **p** | **η²_P_** | **F** | **p** | **η²_P_** | **F** | **p** | **η²_P_** |
| WMC Composite | 1 | 63 | 7.8 | 0.01 * | 0.11 | 9.28 | < 0.01 * | 0.13 | 3.97 | 0.05 | 0.06 |
| Drug | 1 | 63 | < 0.01 | 0.95 | < 0.01 | 0.18 | 0.67 | < 0.01 | 0.66 | 0.42 | 0.01 |
| WMC Composite X Drug | 1 | 63 | 0.07 | 0.79 | < 0.0 | 0.46 | 0.5 | 0.01 | 1.29 | 0.26 | 0.02 |
| Trial-type | 1 | 63 | 69.73 | < 0.01 * | 0.53 | 64.43 | < 0.01 * | 0.51 | 3.58 | 0.06 | 0.05 |
| WMC Composite X Trial-type | 1 | 63 | 0.09 | 0.77 | < 0.01 | < 0.01 | 0.99 | < 0.01 | 0.03 | 0.87 | < 0.01 |
| Trial-type X Drug | 1 | 63 | 4.64 | 0.04 * | 0.07 | 6.25 | 0.02 * | 0.09 | 1.19 | 0.28 | 0.02 |
| WMC Composite X Trial-type X Drug | 1 | 63 | 1.28 | 0.26 | 0.02 | 3.81 | 0.06 | 0.06 | 2.11 | 0.15 | 0.03 |

# Table S3

*F-statistic: Main and interaction effects of 2 X 2 X 2 X 2 factorial repeated measures ANOVA for reaction times (RT).* Within-subjects factors trial-type (Target/Distractor) and drug (L-DOPA/Placebo). Between-subjects factors gender and administration order (L-DOPA first session/L-DOPA second session). Working Memory Capacity (WMC) composite included as covariate. Values rounded to two decimals. DFn = Degrees of Freedom in the numerator, DFd = Degrees of Freedom in the denominator, * Significant, η²_P_ = partial Eta-squared.

|  | **DFn** | **DFd** | **F** | **p** | **η²_P_** |
| --- | --- | --- | --- | --- | --- |
| Gender | 1 | 60 | 0.06 | 0.8 | < 0.01 |
| Administration Order | 1 | 60 | 0.3 | 0.59 | < 0.01 |
| WMC Composite | 1 | 60 | 7.43 | 0.01 * | 0.11 |
| Gender X Administration Order | 1 | 60 | 1.53 | 0.22 | 0.02 |
| Drug | 1 | 60 | 0.01 | 0.94 | < 0.01 |
| Gender X Drug | 1 | 60 | 0.19 | 0.67 | < 0.01 |
| Administration Order X Drug (= Session) | 1 | 60 | 25.53 | < 0.01 * | 0.3 |
| WMC Composite X Drug | 1 | 60 | 0.27 | 0.61 | < 0.01 |
| Gender X Administration Order X Drug | 1 | 60 | 0.29 | 0.59 | < 0.01 |
| Trial-type | 1 | 60 | 38.94 | < 0.01 * | 0.39 |
| Gender X Trial-type | 1 | 60 | 3.03 | 0.09 | 0.05 |
| Administration Order X Trial-type | 1 | 60 | 0.66 | 0.42 | 0.01 |
| WMC Composite X Trial-type | 1 | 60 | 0.11 | 0.75 | < 0.01 |
| Gender X Administration Order X Trial-type | 1 | 60 | 0.67 | 0.42 | 0.01 |
| Drug X Trial-type | 1 | 60 | 5.16 | 0.03 * | 0.08 |
| Gender X Drug X Trial-type | 1 | 60 | 0.42 | 0.52 | 0.01 |
| Administration Order X Drug X Trial-type | 1 | 60 | 3.47 | 0.07 | 0.05 |
| WMC Composite X Drug X Trial-type | 1 | 60 | 0.79 | 0.38 | 0.01 |
| Gender X Administration Order X Drug X Trial-type | 1 | 60 | 0.04 | 0.84 | < 0.01 |

# Table S4

*F-statistic: Main and interaction effects of 2 X 2 X 2 factorial repeated measures ANOVA for reaction times (RT).* Within-subjects factors current trial-type (Target/Distractor), previous trial-type (Target/Distractor) and drug intervention (L-DOPA/Placebo). Values rounded to two decimals. DFn = Degrees of Freedom in the numerator, DFd = Degrees of Freedom in the denominator, * Significant

|  | **DFn** | **DFd** | **F** | **p** |
| --- | --- | --- | --- | --- |
| Drug | 1 | 64 | 0.01 | 0.94 |
| Trial-Type Current Trial | 1 | 64 | 69.06 | < 0.01 * |
| Trial-Type Previous Trial | 1 | 64 | 0.19 | 0.66 |
| Drug X Trial-Type Current Trial | 1 | 64 | 4.49 | 0.04 * |
| Drug X Trial-Type Previous Trial | 1 | 64 | 0.12 | 0.73 |
| Trial-Type Current Trial X Trial-Type Previous Trial | 1 | 64 | 0.04 | 0.85 |
| Drug X Trial-Type Current Trial X Trial-Type Previous Trial | 1 | 64 | 5.8 | 0.02 * |


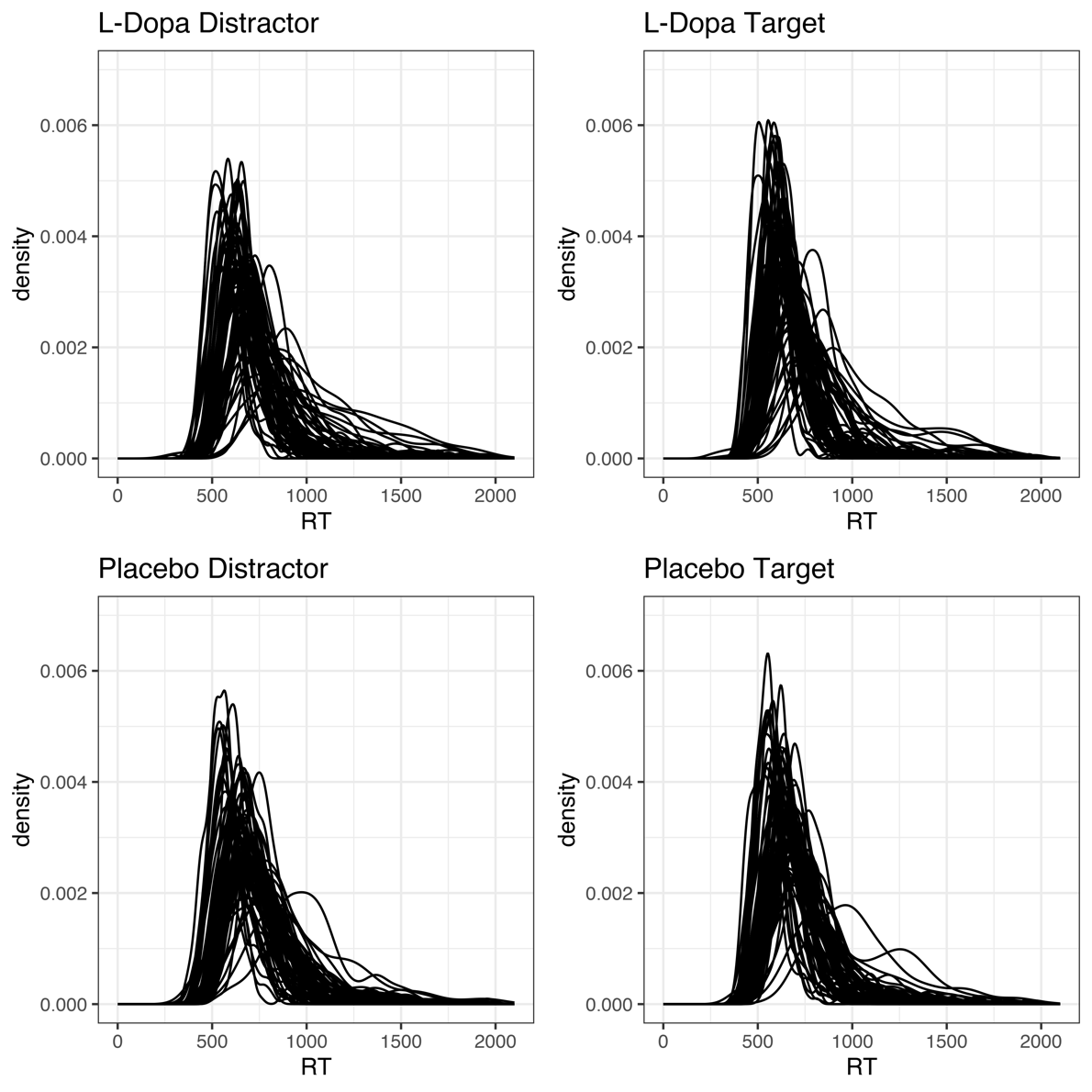


# Fig. S1

*Density Plot of RT Distribution in Each Individual Participant.* Density curves of RTs (in ms) are depicted for each participant across drug (L-DOPA/Placebo) and trial-type (Target/Distractor).


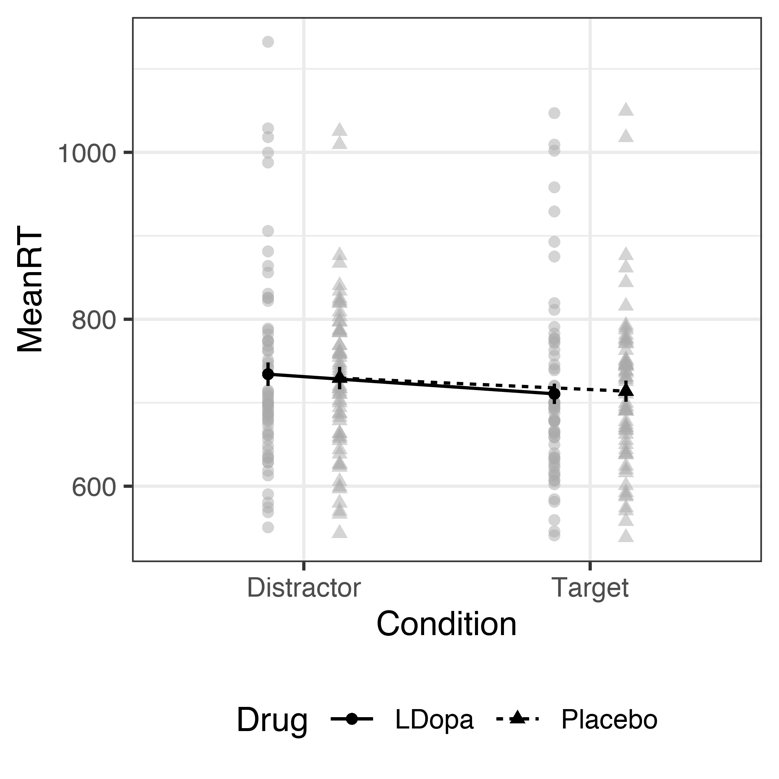


# **Fig. S2**

*Effects of Drug and Trial-Type on Reaction Time (RT).* Presented are the individual RT data with means across drug (L-DOPA/Placebo) and trial-type (Target/Distractor). The RT distractor effect (RT_Distractor-Trials_ – RT_Target-Trials_) was more pronounced during the L-DOPA session compared to the Placebo session. There was high inter-individual variance in RTs.


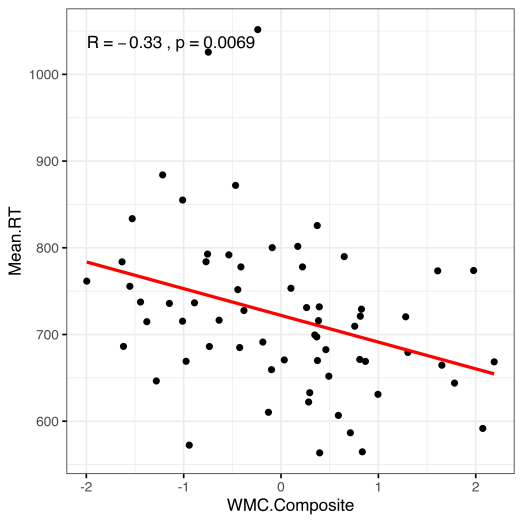


# **Fig. S3**

*Association of Reaction Time (RT) and Working Memory Capacity*. There was a significant linear relationship between the working memory capacity (WMC) composite score and RTs regardless of trial-type and drug.


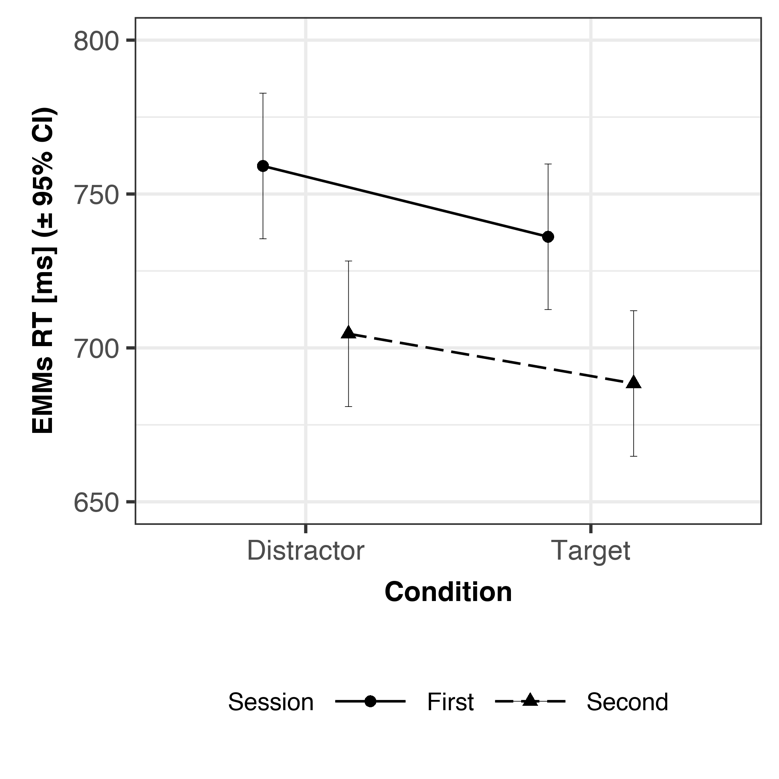


# **Fig. S4**

*Effects of Session and Trial-Type on Reaction Time (RT).* Presented are the estimated marginal means (EMM) across session (First/Second) and trial-type (Target/Distractor). Participants performed faster in the second session across trial-types. There was a statistical trend for a smaller RT distractor effect (RT_Distractor-Trials_ – RT_Target-Trials_) during the second session compared to the first session.


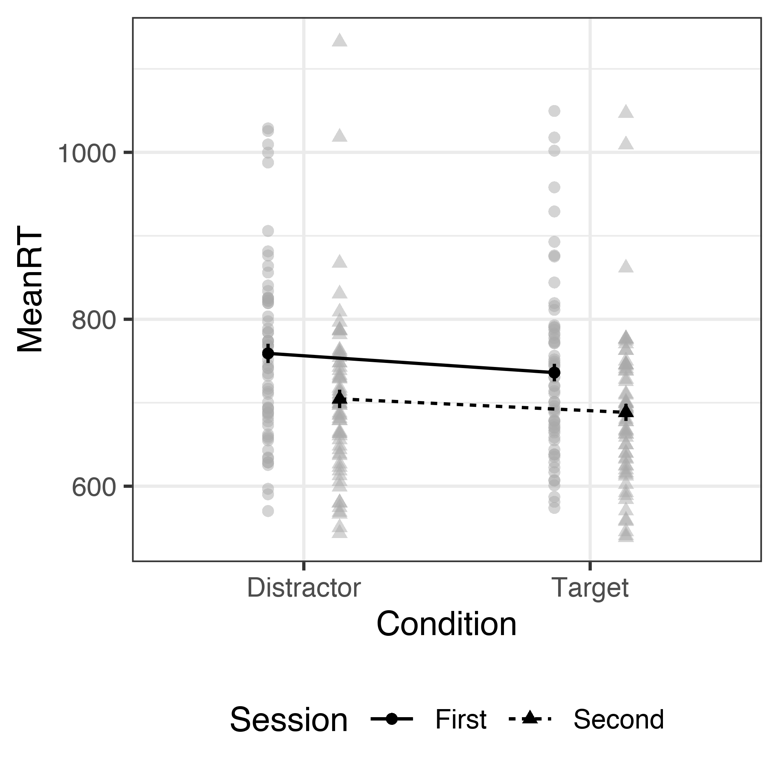


# **Fig. S5**

*Effects of Session and Trial-Type on Reaction Time (RT).* Presented are the individual RT data with means across session (First/Second) and trial-type (Target/Distractor). Participants performed faster in the second session across trial-type. There was a statistical trend for a smaller RT distractor effect (RT_Distractor-Trials_ – RT_Target-Trials_) during the second session compared to the first session. There was high interindividual variance in RTs.

**
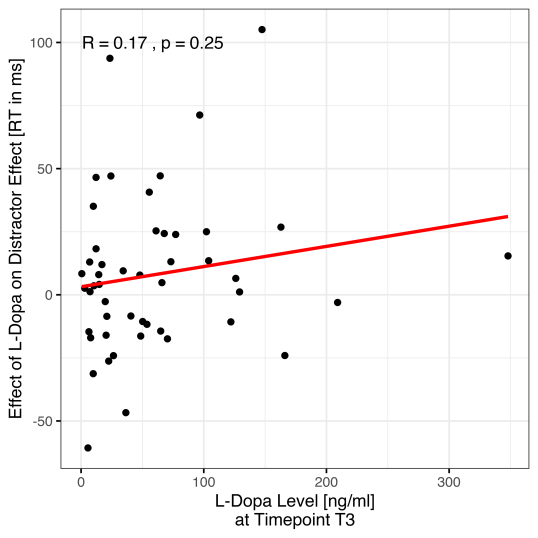
**

# **Fig. S6**

*Association of L-DOPA-induced Change in the RT Distractor Effect and L-DOPA Serum Levels.* Presented are the L-DOPA-induced change in the RT distractor effect [(RT_Distractor-Trials-L-DOPA_ – RT_Target-Trials-L-DOPA_)- (RT_Distractor-Trials-Placebo_ – RT_Target-Trials-Placebo_)] with respect to the individual L-DOPA serum levels at timepoint T3 (40 min after booster dose of 75 mg L-DOPA, 35 to 50 min before the VST) in the verum session. There was no statistically significant linear relationship. The direction of the association is in line with an expected stronger modulation with higher L-DOPA serum levels.

**
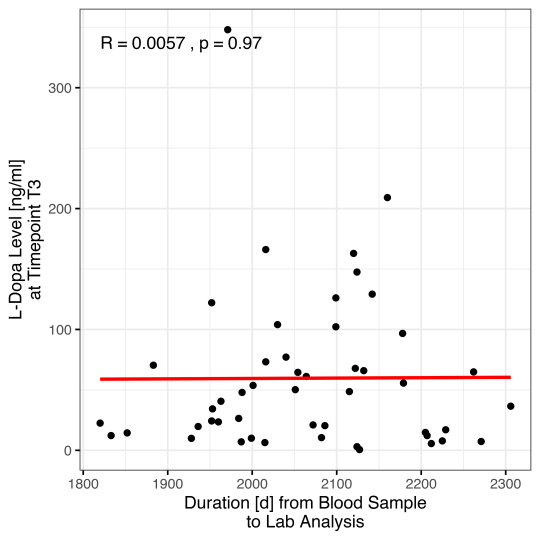
**

# **Fig. S7**

*Association of L-DOPA Serum Levels and Time from Blood Sampling to Laboratory Analysis*. Presented are the individual L-DOPA serum levels with respect to the days (d) that past between the time the blood was collected to when it was analysed in the laboratory. There was no statistically significant linear relationship. That is, lower than expected L-DOPA serum levels were statistically not associated with longer storage before analysis.


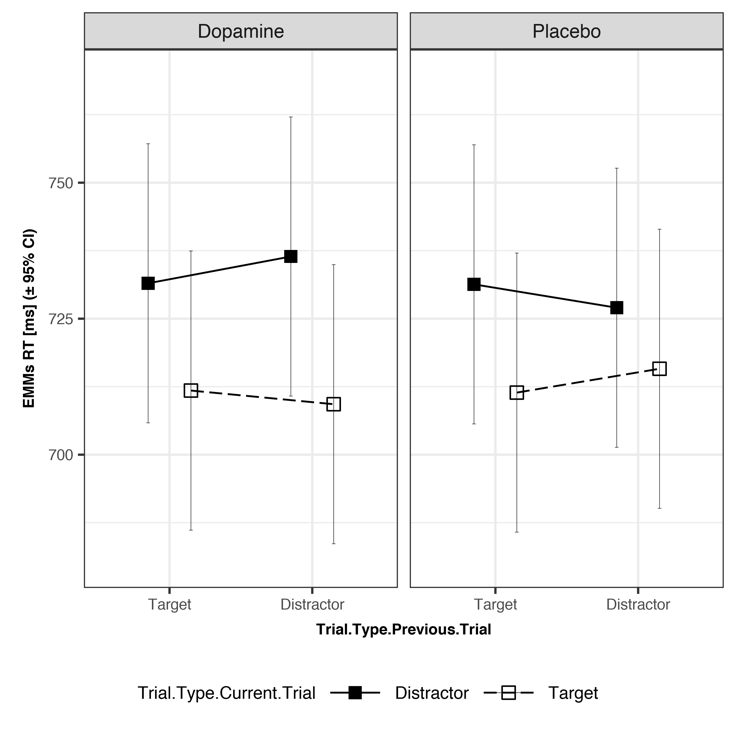


# **Fig. S8**

*Effects of Drug, Current Trial-type (Target/Distractor), and Previous Trial-type (Target/Distractor) on Reaction Time (RT).* Presented are the estimated marginal means (EMM) across current trial-type (Target/Distractor) and previous trial-type (Target/Distractor) for the L-DOPA session on the left and the Placebo session on the right panel. It is illustrated that the L-DOPA induced increase of the RT distractor effect was clearly present for trials that followed a distractor trial (compare right column in each panel), but was not detectable for trials that followed a target trial (compare left column in each panel). Numerically, participants responded slower on distractor trials that followed a distractor trial in the L-DOPA session compared to the placebo session. At the same time, participants responded faster (numerically) on target trials that followed a distractor trial in the L-DOPA session compared to the placebo session.


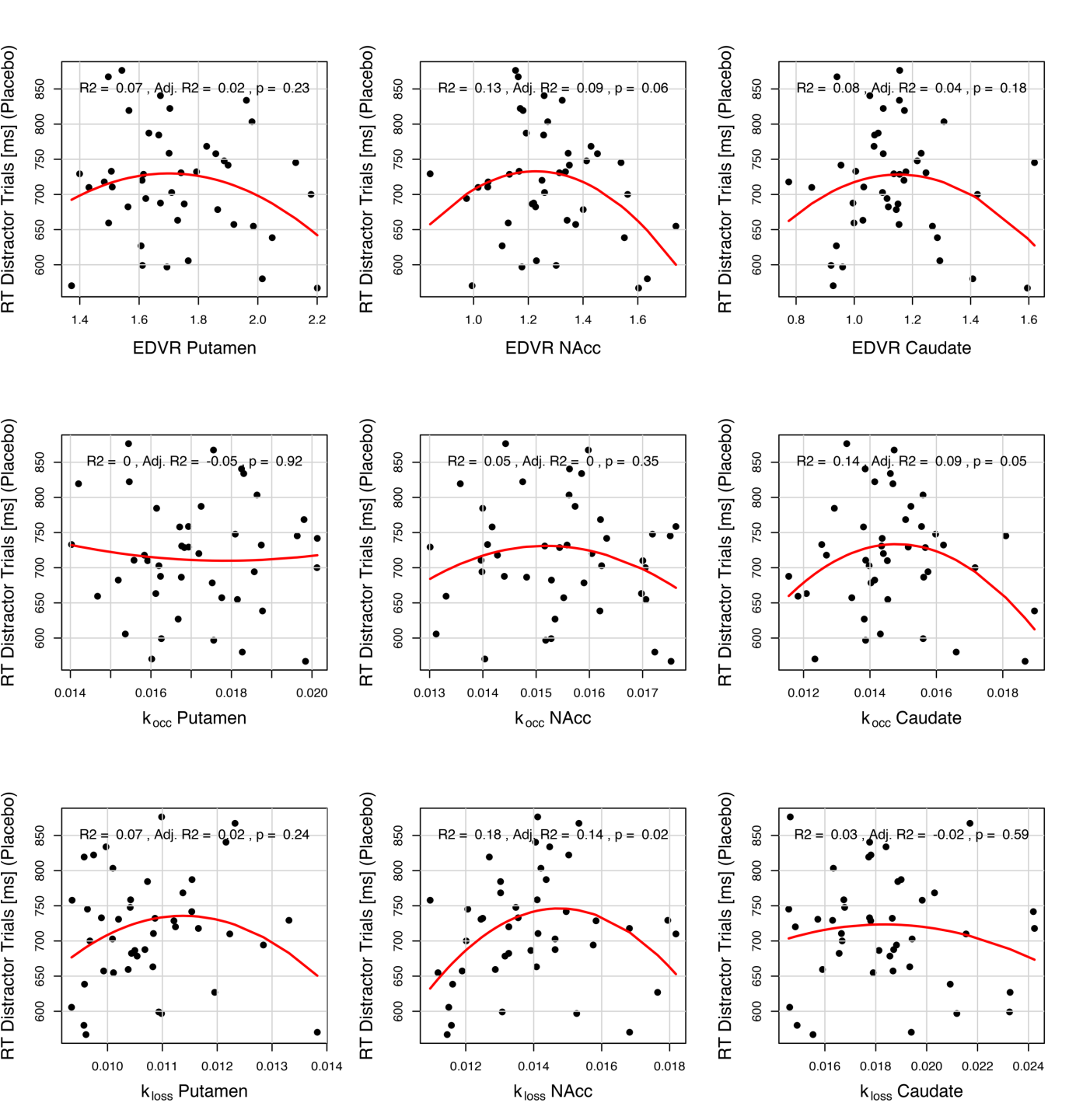


# **Fig. S9**

*Association of the Mean RT in Distractor Trials (Placebo) and Baseline Striatal Tonic Dopamine*. There was no quadratic model fit between any of the ^18^F-DOPA-PET measures (*k*_occ_, EDVR, *k*_loss_) in any striatal ROI (putamen, N_acc_, caudate) and RTs on distractor trials in the placebo session that would have been significant after correction for multiple comparisons. *k*_occ_ = influx rate constant, EDVR = effective distribution volume ratio, *k*_loss_ = washout rate, N_acc_ = Nucleus accumbens.
